# Hippocampal stem cells promotes synaptic resistance to the dysfunctional impact of amyloid beta oligomers via secreted exosomes

**DOI:** 10.1186/s13024-019-0322-8

**Published:** 2019-06-14

**Authors:** Maria-Adelaide Micci, Balaji Krishnan, Elizabeth Bishop, Wen-Ru Zhang, Jutatip Guptarak, Auston Grant, Olga Zolochevska, Batbayar Tumurbaatar, Whitney Franklin, Claudia Marino, Steven G. Widen, Arjun Luthra, Steven G. Kernie, Giulio Taglialatela

**Affiliations:** 1 Department of Anesthesiology, University of Texas Medical Branch, Galveston, TX 77555 USA; 2 Mitchell Center for Neurodegenerative Diseases, Department of Neurology, University of Texas Medical Branch, Galveston, TX 77555 USA; 3 Biochemistry & Molecular Biology, University of Texas Medical Branch, Galveston, TX 77555 USA; 4 Pressent address: The University of Texas at Austin, Austin, TX 78712 USA; 5 Department of Pediatrics and Pathology & Cell Biology, Columbia University College of Physicians and Surgeons, New York, NY 10032 USA

**Keywords:** Alzheimer’s disease, Aβ oligomers, Neural stem cells, Exosomes, Synapses

## Abstract

**Background:**

Adult hippocampal neurogenesis plays an important role in synaptic plasticity and cogntive function. We reported that higher numbers of neural stem cells (NSC) in the hippocampus of cognitively-intact individuals with high Alzheimer’s disease (AD) pathology (plaques and tangles) is associated with decreased synaptic amyloid beta oligomers (Aβο), an event linked to onset of dementia in AD. While these findings suggest a link between NSC and synaptic resistance to Aβο, the involved mechanism remains to be determined. With this goal in mind, here we investigated the ability of exosomes secreted from hippocampal NSC to promote synaptic resilience to Aβo.

**Methods:**

Exosomes isolated from media of hippocampus NSC (NSC-exo) or mature hippocampal neuronal (MN-exo) cultures were delivered intracerebroventricularly (ICV) to mice before assessment of Aβο-induced suppression of hippocampal long-term potentiation (LTP) and memory deficits. Aβο binding to synapses was assessed in cultured hippocampal neurons and on synaptosomes isolated from hippocampal slices from wild type mice and from an inducible mouse model of NSC ablation (Nestin-δ-HSV-TK mice) treated with exosomes. Expression of CaMKII and of AMPA and NMDA glutamate receptor subunits in synaptosomes was measured by western blot. Small RNA Deep sequencing was performed to identify microRNAs enriched in NSC-exo as compared to MN-exo. Mimics of select miRNAs were injected ICV.

**Results:**

NSC-exo, but not MN-exo, abolished Aβo-induced suppression of LTP and subsequent memory deficits. Furthermore, in hippocampal slices and cultured neurons, NSC-exo significantly decreased Aβo binding to the synapse. Similarly, transgenic ablation of endogenous NSC increased synaptic Aβo binding, which was reversed by exogenous NSC-exo. Phosphorylation of synaptic CaMKII was increased by NSC-exo, while AMPA and NMDA receptors were not affected. Lastly, we identified a set of miRNAs enriched in NSC-exo that, when injected ICV, protected the synapses from Aβo-binding and Aβo-induced LTP inhibition.

**Conclusions:**

These results identify a novel mechanism linking NSC-exo and synaptic susceptibility to Aβo that may underscore cognitive resilience of certain individuals with increased neurogenesis in spite of AD neuropathology and unmask a novel target for the development of a new treatment concept for AD centered on promoting synaptic resilience to toxic amyloid proteins.

**Electronic supplementary material:**

The online version of this article (10.1186/s13024-019-0322-8) contains supplementary material, which is available to authorized users.

## Background

Generation of new neurons from neural stem cells (NSC) has been shown to occur in the dentate gyrus (DG) of the hippocampus [1], a brain region involved in learning and memory and most affected in AD [2–5]. Although recent reports have re-ignited the discussion on the extent of proliferation of NSC and number of newly generated neurons in the human hippocampus [6, 7], there is ample consensus that neurogenesis contributes significantly to synaptic plasticity and cognitive function [8]. Specifically, reports have shown that while decreased numbers of NSC in the DG result in impairments in learning and memory [9–11], factors known to increase NSC numbers and neurogenesis, such as exercise and environmental enrichment, result in improved learning and memory [12–14]. Notably, impaired neurogenesis is believed to be a key-contributing factor to AD pathology-driven neuronal dysfunction [15–18].

In the past two decades, the discovery of the existence of individuals who remain cognitively intact despite the presence of neuropathological features (amyloid plaques and tau tangles) normally associated with a fully symptomatic stage of AD (hereafter referred to as Non-Demented with AD Neuropathology -- NDAN) has significantly impacted the field by revealing that there is a natural way for the brain to resist (or significantly delay) the neurotoxic events that normally lead to cognitive demise in AD (reviewed in Zolochevska and Taglialatela, 2016) [19]. We previously reported that hippocampal synapses in NDAN subjects display a unique proteomic profile [20] and are resistant to the disabling binding of toxic oligomers of amyloid β (Aβo) [21]. This is significant in that targeting of synapses by toxic Aβ and tau oligomers is reputed to be an early key event in AD, resulting in synaptic disruption that initially drives onset and progression of cognitive decline [22–26]. Therefore, preventing synaptic disruption by amyloid oligomers, as seen in the cognitively intact NDAN subjects, would be an effective therapy for AD. However, a strategy to achieve such a therapeutic objective remains elusive.

In the pursuit of unveiling such a strategy, we have previously reported that NDAN individuals present with a significantly increased number of NSC in the hippocampus DG, as compared to both AD patients and control subjects, and that this increased number of NSC positively correlates with cognitive function [27]. While this was the first report demonstrating such a correlation in the human brain, it was consistent with numerous literature evidences in animal models showing that an increased number of NSC is associated with improved learning and memory [12–14]. Notably, however, contrary to the number of NSC, the number of mature neurons in the DG of NDAN individuals did not correlate with preserved cognition [27], suggesting that the ability of NSC to support cognitive resilience in NDAN is mediated by a mechanism other than their capacity to generate new neurons. While these observations suggest a link between increased NSC numbers and synaptic resistance to Aβ oligomers toxicity, the involved mechanism remains to be determined.

NSC are known to release large amounts of exosomes [28, 29] and recent literature indicates that NSC-derived exosomes (NSC-exo) mediate many of the functional effects classically attributed to these cells [30–32]. Exosomes are small (40–100 nm diameters), secreted vesicles containing cell-specific cargos of proteins, lipids and, most abundantly, regulating microRNAs (miRNAs) [33, 34]. Secreted exosomes are taken up by target cells where they deliver their cargo which modulate cells’ function and physiological homeostasis [35]. Exosomes have been shown to mediate the control of ageing speed exerted by hypotalamic NSC [32] and to ameliorate synaptic dysfunction and cognitive decline in the APP/PS1 transgenic AD mouse model [36]. Therefore, signaling via exosome release is emerging as a key feature of NSC, mediating their effects on synaptic and cognitive function and involved in neurodegenerative disorders such as AD. In addition, a recent meta-analysis of comprehensively curated datasets from current relevant literature has shown that exosomes and homeostatic synaptic plasticity are linked to each other and, together, to several neurodegenerative diseases including Huntington’s, Parkinson’s and AD [37].

Based on this evidence, in the present work we investigated whether exosomes secreted by NSC prevent Aβ oligomer-driven functional synaptic deficits and memory impairments and whether the mechanism underlying these protective effects by NSC-exo involves increased synaptic resistance to Aβ oligomer binding. Moreover, because miRNAs represent the most abundant exosomal cargo and have been shown to mediate many of the effects elicited by exosomes, including modulation of aging [32], cognition and synaptic function [38–40] and neuroprotection [41], we performed deep sequence analysis to identify select miRNAs uniquely present in NSC-exo (as compared to MN-exo) that could potentially mediate their protective action on synapses of target neurons.

## Methods

### Animals

Mice (C57BL/6 J; 6–8 weeks old) were purchased from Jackson Laboratories (Bar Harbor, ME). Nestin-δ-HSV-TK mice were generated as previously described [42]. Both males and females mice were used in these studies.

### Valganciclovir treatment

Valganciclovir powder was mixed into mouse chow (0.09%, Valcyte, valganciclovir hydrochloride) and given ad libitum for an average dose of ~ 90 mg/kg/day (Custom Animal Diets, LLC, Easton, PA). Animals were fed either Valganciclovir-containing chow or standard chow (“vehicle”) right after weaning for 4 weeks.

### Neural stem cells and mature neurons cultures

Adult rat hippocampus-derived NSC were purchased from MilliporeSigma (Tenecula, CA). NSC were cultured as neurospheres in low-attachment plates in expansion media consisting of: EmbryoMax DMEM/F12 with L-Glutamine, without HEPES, B27-with retinoic acid, GlutaMAX™ (all from Gibco, ThermoFisher Scientific, Waltham, MA), FGF-b (20 ng/ml, MilliporeSigma, Tenecula, CA) and antibiotics (PSF, Gibco, ThermoFisher Scientific, Waltham, MA). Cells were passaged every 5–7 days using Neuropapain (Genlantis, San Diego, CA). For the generation of mature neurons, NSC neurospheres were dissociated using Neuropapain (Genlantis, San Diego, CA), 2 mg/ml in basal media, plated out onto poly-ornithine/laminin-coated plates at the density of 8.0 × 10^5^ cells/cm^2^ and cultured in differentiation media consisting of expansion media without FGFb and with the addition of 1 μM retinoic acid (MilliporeSigma, Tenecula, CA) and 5 μM forksolin (MilliporeSigma, Tenecula, CA) for 5 days.

### Western blotting analysis of neural stem cells and mature neurons

Cells growing as neurospheres in complete growth media were collected in a conical tube and centrifuged at 220 g for 5 min at room temperature. The cells were washed in Dulbecco’s PBS and lysed in RIPA buffer containing protease inhibitors cocktail (200 μl of lysis buffer for < 5 × 10^6^ cells). The lysed cells were transferred to 1.5 ml Eppendorf tubes, vortexed and incubated on ice for 20 min. Total protein content was measured using Pierce BCA Protein Assay Kit (Thermo Fisher Scientific, Rockford, IL) and stored at  -20 °C. Cells growing on poly-ornithine/laminin-coated plates were detached using StemPro Accutase, collected into 15 ml tubes and centrifuged at 220 g for 5 min. The cell pellet was washed in Dulbecco’s PBS and lysed in RIPA lysis buffer and total protein content was measured using the Pierce BCA protein assay kit. The protein samples were processed for SDS-polyacrylamide gel electrophoresis (PAGE) with Mini-PROTEAN Tetra Cell System (Bio-Rad, Hercules, CA). After electrophoresis, proteins were transferred to Polyvinylidene Difluoride (PVDF) membranes (Bio-Rad) overnight at 4 °C. Blots were incubated in blocking buffer (5% non-fat dry milk in TBS containing 0.1% Tween 20; TBS-T) for 1 h at room temperature and probed with primary antibodies diluted in blocking buffer [1:1000] overnight at 4 °C (Sox2, Cell Signaling Technology; Nestin, Millipore; NeuN, CellSignaling; βIII-tubulin, Promega). The membranes were subsequently washed and then incubated for 1 h with an anti-mouse or anti-rabbit IgG horseradish peroxidase-linked secondary antibody [1:5000] (Cell Signaling Technology). GAPDH was used as a loading control for whole-cell lysate (Invitrogen). Membranes were visualized by incubating for 2 min in SignalFire Plus ECL Reagent (Cell Signaling Technology, Danvers, MA, USA) followed by chemiluminescence detection on ChemiDoc XRS+ System, Image Lab Software (Bio-Rad, Hercules, CA).

### Amyloid β oligomers preparation

Human amyloid β (Aβ_1–42_ peptide) was purchased from the Department of Biophysics and Biochemistry of Harvard University (MA). Amyloid β oligomers were prepared according to established methods [43–45]. Briefly, lyophilized synthetic Aβ aliquots (0.3 mg) were dissolved in 0.2 ml of 1,1,1,3,3,3- Hexafluro-2-propanol (HFP) and subsequently diluted using 700 μl of H_2_O. A cap with four holes was placed on the tube and the sample was stirred by a magnetic stir bar under a fume hood for 48 h. The sample was used immediately after the 48 h of stirring [43]. Western blot and dot blot analyses using Aβ oligomer specific A-11 antibodies were used to determine the quality of oligomerization as previously described [46] (data not shown).

For the flow cytometry analysis of Aβ binding to synaptosomes and in vitro binding to cultured hippocampal neurons, fluorescently tagged Aβ oligomers were prepared by adding Aβ_1–42_ peptide (HiLyteTM Fluor 647 or HiLyteTM Fluor 488, AlexaFluor, AnaSpec Inc.) to HFP-Aβ oligomers. This mixture was then added to 0.7 ml of H_2_O and stirred for 48 h as described previously [46, 47].

### Exosomes isolation and characterization

Exosomes were isolated from conditioned culture media using the ultracentrifugation method [48, 49]. Briefly, 225 ml of conditioned media collected from approximately 60 million cultured cells (NSC or mature neurons) were centrifuged at 2000 x g, at 4 °C for 10 min to remove cells and debris. The resulting supernatant was transferred to new tubes and centrifuged at 10,000 x g, at 4 °C for 10 min. The supernatant was transferred to Beckman 60 Ti ultracentrifuge tubes and centrifuged at 126,000 x g for 3 h at 4 °C. The resulting pellet was resuspended in 2 ml PBS containing a protease inhibitor cocktail (MilliporeSigma, Tenecula, CA), and centrifuged at 135,000 x g for one hour at 4 °C in a Beckman TLA110 centrfuge. The resulting pellet, containing exosomes, was resuspended in 1X PBS containing protease inhibitors to a concentration of approximately 10^9^ exosomes per microliter.

Comprehensive characterization of isolated exosomes was performed using electron microscopy (EM), western blotting (WB) and nanoparticle tracking analyses. For EM, approximately 5 μl of exosome preparation was incubated on carbon film grid (200 mesh, copper), washed, and stained with uranyl acetate. Images were acquired on a Philips CM-100 transmission electron microscope at 60 kV with an Orius SC2001 digital camera (Gatan, Pleasanton, CA). Western blot was performed using 20 μl exosome preparation lysed with RIPA buffer containing protease inhibitors. The proteins were separated on a SDS-PAGE gel and transferred to a nitrocellulose membrane. The membrane was probed with primary antibodies against CD9, CD63, CD81, Hsp70 and GM130 (SBI Biotechnology) overnight at 4 °C, followed by IR Dye® 680 nm and 800 nm secondary antibodies based imaging and quantification (LI-COR Biosciences). Nanoparticle tracking analysis was performed using the NanoSight N300 system and NTA 2.1 operating system (Malvern) according to manufacturer’s instructions. Briefly, the exosome preparation was diluted in PBS and injected into the NanoSight for analysis of both particle size and concentration.

### Intracerebroventricular (ICV) injections

Adult (6–8 weeks old) C57Bl/6 J mice were anesthetized with isoflurane and subjected to ICV injections using the freehand injection method [45, 50, 51]. Briefly, a 29-gauge needle, firmly held with hemostatic forceps to leave 4.5 mm of the needle tip exposed, was connected to a 25 μl Hamilton syringe via 0.38 mm polyethylene tubing. Infusions were performed at the rate of 3 μl/min for a total volume of 3 μl, using an electronic programmable microinfuser (Harvard Apparatus). After ICV injection, the needle was left in place for a minute and the mouse was allowed to recover while lying on a heated pad under warm light.

One day after ICV injection of exosomes (prepared from NSC or MN conditioned media) or PBS (vehicle), mice were treated ICV with Aβο (prepared as described above) and euthanized 24 h later (for biochemistry and electrophysiology studies) or 48 h later (at the end of novel object recognition testing) by using deep anesthesia with isoflurane. The brains were removed and processed for fresh hippocampal slices preparation (described in detail below), or further dissected into hippocampi, frontal cortex, parieto-occipital cortex and midbrain, snap frozen on dry ice and stored at − 80 °C until ready to use for synaptosomes preparation.

### Brain slices preparation and electrophysiological assessments of long-term potentiation

Brain slices were prepared according to the method reported by Ting et al. [52] and described below. Mice were deeply anesthetized with isoflurane and transcardially perfused with 25–30 mL of room temperature carbogenated (95% O_2_ and 5% CO_2_ gas mixture) NMDG ACSF consisting of 92 mM NMDG, 2.5 mM KCl, 1.25 mM NaH_2_PO_4_, 30 mM NaHCO_3_, 20 mM HEPES, 25 mM glucose, 2 mM thiourea, 5 mM Na-ascorbate, 3 mM Na-pyruvate, 0.5 mM CaCl_2_·4H_2_O and 10 mM MgSO_4_·7H_2_O, titrated to pH 7.3–7.4 with HCl. Following perfusion the brains were gently extracted from the skull within one minute. A transverse slicing angle was used to block the brain using super glue to the mounting cylinder to generate slices using the Compresstome VF-300 (Precisionary Instruments) protocols, to get 350 μM sections. Using a cut-off plastic Pasteur pipet, an initial protective recovery was done in the cutting solution at 32–34 °C for < 12 min. The slices were then transferred to a new holding chamber with carbogen saturated HEPES ACSF recovery solution (92 mM NaCl, 2.5 mM KCl, 1.25 mM NaH_2_PO_4_, 30 mM NaHCO_3_, 20 mM HEPES, 25 mM glucose, 2 mM thiourea, 5 mM Na-ascorbate, 3 mM Na-pyruvate, 2 mM CaCl_2_·4H_2_O and 2 mM MgSO_4_·7H_2_O) at room temperature. Recording was done in constantly flowing oxygenated (95% O_2_/5% CO_2_) ice-cold normal artificial cerebrospinal fluid (nACSF) consisting of (in mM) 125 NaCl, 2.5 KCl, 1.25 NaHPO_4_, 25 NaHCO_3_, 2 CaCl_2_, 1.3 MgCl_2_, and 10 dextrose, pH 7.3. Slices were perfused at a rate of approximately three mL/min. Field excitatory post-synaptic potentials (fEPSPs) were collected using an Axon MultiClamp 700B amplifier connected to a Windows computer running Clampex 8.2 software (Molecular Devices). All electrodes were placed under the visual guidance of an upright microscope (Nikon).

The slope from a single fEPSP trace was calculated from the initial slope of the fEPSP relative to the slope of the 10 milliseconds interval immediately preceding afferent stimulation. The current magnitude was delivered through a digital stimulus isolation amplifier (A.M.P.I) and set to elicit a fEPSP approximately 30% of maximum for synaptic potentiation experiments using platinum-iridium tipped concentric bipolar electrodes (FHC Inc). Using a horizontal P-97 Flaming/Brown Micropipette puller (Sutter Instruments), borosilicate glass capillaries were used to pull electrodes and filled with nACSF to get a resistance of 1–2 MΩ. A stable baseline (for a minimum of ten min) was obtained by delivering single pulse stimulation at 20 s interstimulus intervals. fEPSPs in the CA1 were evoked by stimulating the Schaffer collaterals (SC: CA3- > CA1) using a conditioning stimulus (CS) consisting of three trains of 100 pulses at 100 Hz, twenty seconds apart (high-frequency stimulation; HFS). Input-output experiments were conducted to measure basal dendritic excitation in response to increasing applied current in nACSF. Evoked fEPSP responses were digitized via Digidata 1550B and the initial slope of the fEPSP was analyzed using pClamp 10.6 software (Molecular Devices). All data are represented as percentage change from the initial average baseline fEPSP slope, which was defined as the average slope obtained for the ten minutes prior to CS application.

### Novel object recognition (NOR) testing

NOR testing was performed as described previously [47, 50, 53]. Briefly, each mouse was habituated to an empty novel object recognition open field box that served to test each animal for normal locomotion. The sessions commenced with two 10 min habituation sessions spaced 24 h apart, during which the TopScan (Clever Sys. Inc., Reston, VA) video-tracking software quantifies various locomotor parameters. Twenty-four hours after the last habituation session, mice were subjected to training in another 10 min session of exposure to two identical, non-toxic objects (metal or hard plastic items) in the acclimated open field box, so that the animals spent maximum amount of time exploring the objects. The time spent exploring each object was recorded using ObjectScan (Clever Sys. Inc.) by determining a quadrant zone and object zones surrounding the object. After the training session, the animal was returned to its home cage and after a variable retention interval of two hour to twenty-four hours, the animal was returned to the arena in which two objects, one identical to the familiar object but previously unused (to prevent olfactory cues and prevent the necessity to wash objects during experimentation) and one novel object. The animal was allowed ten minutes, during which the amount of time exploring each object was again recorded. Objects were randomized and counterbalanced across animals. The ratio of the difference in time spent exploring each object (familiar versus novel) to the sum of the two was reported as an object discrimination index (ODI). An index above 0.5 is indicative of novelty associated with the object. Each mouse was tested at 2 h and at 24 h with the intention of assessing the shorter and longer time frames in memory recall. In order to avoid that the experience in the 2 h test would affect performance in the 24 h test, different novel objects were used for the two memory recall tests.

### Synaptosomes preparation

Synaptosomes were isolated using a standardized method developed in the lab [54] and routinely used to assess synaptic dysfunction in our studies [47, 50]. Briefly, brain tissue (snap frozen brain regions: hippocampus, frontal cortex, parieto-occipital cortex and midbrain) [55] or freshly prepared brain slices were homogenized in Syn-PER synaptic protein extraction reagent (ThermoFisher Scientific, Waltham, MA) containing 1X Protease Inhibitor Cocktail (Sigma-Aldrich) and Halt Phosphatase Inhibitor Cocktail (Life Technologies, Inc). A portion of the total homogenate was saved for biochemical analysis and the remaining portion was centrifuged at 1200×g for 10 min at 4 °C. The supernatant was collected and centrifuged further at 15,000×g for 20 min at 4 °C. The synaptosomes (contained in the pellet) were resuspended in HEPES-buffered Krebs-like (HBK) buffer (for electron microscopy, ICV and flow cytometry studies) or radioimmunoprecipitation assay (RIPA) buffer (for WB and ELISA studies).

Ultrastructure analysis of synaptosomes was done on 5 μL drop adsorbed on 200 mesh coated resin grid (FCF 200 – CU Formavar/Carbon, Electron Microscopy Sciences) and stained with 2% aqueous uranil acetate for negative staining. Both before and after staining, adsorbed samples were washed three times with DDI water 0.2 μm filtered. Resins were blotted with filter paper and dried with warm regular light. Acquisitions were done using a J EM- 1400 80 KV (Jeol) (Additional file 1: Figure S1A). Synaptosomes were counted using flow cytometry (Guava Easy Cyte 8, Millipore) and correct size between 1 μm and 5.6 μm for the analysis was chosen using standard size beads as a reference (EMD Millipore) (Additional file 1: Figure S1B).

### Quantification of Aβ oligomers binding to synaptosomes

#### ELISA

The Human Aβ42 Ultrasensitive ELISA Kit (Invitrogen, Carlsbad, CA), recognizing natural and synthetic human Aβ42, was used to detect Aβ oligomers in isolated synaptosomes. 15,000 synaptosomes or ~ 0.03 μg protein was loaded per well**.** ELISA was performed according to manufacturer’s instructions.

#### Flow cytometry

To determine the amount of Aβ oligomers associated with the synaptosomes, two million synaptosomes were incubated with 2.5 μM HiLyteTM Fluor 647-labeled Aβ oligomers (HiLyteTM Fluor 647-labeled Aβ – Anaspec Inc., Fremont, CA; Aβ oligomers – Department of Biophysics and Biochemistry, Yale University, New Haven, CT) for 1 h at room temperature in dark. The samples were washed three times in HBK buffer to remove all unbound Aβ oligomers and resuspended in PBS without Ca^2+^/Mg^2+^. The samples were analyzed using Guava easyCyte flow cytometer (MilliporeSigma, Burlington, MA). Standard size polystyrene particles (Spherotech, Inc., Lake Forest, IL) were used to set up size 1–5 μm gate for synaptosomes analyses.

#### Western blotting analysis of synaptosomes

Isolated synaptosomes were resuspended in RIPA buffer, protein concentration was measured using BCA (Thermo Fisher Scientific, Waltham, MA). Separation of the proteins in the samples obtained was done by 8% SDS- polyacrylamide gel electrophoresis. The separated proteins were transferred to a nitrocellulose membrane (Bio-Rad Laboratories, Hercules, CA) and incubated with several antibodies: pGluR1, pGluR2 (1:1000, Abcam, Cambridge, UK); GluR1, NR2B, GluR2, NR1 (1:1000, NeuroMab, UCDavis, CA); pNR2B, pNR1 (1:1000, MilliporeSigma, Burlington, MA); CaMKII, pCaMKII (1:1000, Santa Cruz Biotechnology, Inc., Dallas, TX). β-actin (1:3000, MilliporeSigma, Burlington, MA) was used as a loading control. The membrane was incubated with proper fluorescent secondary antibodies (1:10,000) (LI-COR Biosciences, Lincoln, NE) and scanned using Odyssey infrared fluorescent imaging system (LI-COR Biosciences, Lincoln, NE).

#### Aβ oligomer binding to hippocampal neurons in vitro

Hippocampal neurons were generated by differentiating adult hippocampal NSC as described above and plated on poly ornithine/laminin-coated plates. Cultures were treated with exosomes (NSCexo or MNexo) or equivalent volume of PBS for 24 h at 37 °C. After washing, cultures were exposed to 2.5 μM Fluor 647-Aβo or vehicle (PBS) for 30 min at 37 °C. At the end of the incubation, the cells were washed and fixed in 4% paraformaldehyde for 15 min. After two washes in PBS, the cells were blocked and permeabilized in PBS containing 5% normal goat serum for 30 min at room temperature. The cells were incubated with mouse anti-βIII-tubulin primary antibody (1:1000, Promega, Madison, WI) diluted in 1.5% normal goat serum in PBS overnight at 4 °C in a humid chamber. Following 3 washes in PBS, the cells were incubated with Alexa 488-conjugated anti-mouse secondary antibody (1:400; Invitrogen, Carlsbad, CA) in 1.5% normal goat serum in PBS for 1 h at room temperature in a humid chamber. The cells were washed in PBS and coverslipped with Prolong® Gold AntiFade Reagent with DAPI (ThermoFisher Scientific, Waltham, MA). Images were acquired with an Olympus confocal microscope (FV1200, Olympus Life Science) and quantification of Aβo binding was performed by an investigator blinded to the experimental groups by counting the number of fluorescent puncta in dendrites using ImageJ software.

#### Exosomes labelling

Exosomes were labeled using the PKH26 Red Fluorescent Cell Linker Kit (Sigma-Aldrich, St. Louis, MO). Briefly, 2 μM PKH26 was added to the exosomes suspension and incubated for 4 min under sterile conditions. After the addition of 1% BSA/PBS, the exosomes were centrifuged at 135,000 x g for one hour at 4 °C in a Beckman TLA110 and resuspended in PBS and used for subsequent experiments. PKH26-labeled exosomes were injected ICV (as described above) in adult wild type mice (for in vivo tracking) or used for in vitro binding experiments with Fluor 488-labelled Aβo.

#### Tissue processing and immunofluorescence analysis

For localization of labelled exosomes in the brain, 4 h after ICV injection, mice were deeply anesthetized using isoflurane and euthanized. The brains were removed, fresh frozen over dry ice and embedded in O.C.T. compound (Tissue-Tek; Tokyo, Japan). Sections (10 μm thick) were cut on a cryostat (Leica CM 3050S), collected onto Superfrost/Plus slides (ThermoFisher Scientific, Waltham, MA) and stored at − 80 °C until use. For immunofluorescence, slides were removed from − 80 °C and fixed in 4% paraformaldehyde (PF) in 0.1 M PBS, pH 7.4 for 15 min at room temperature, blocked and permeabilized with 5% normal goat serum /0.3%Triton X-100/0.05% Tween-20 in PBS, and incubated with rabbit anti-NeuN primary antibody (1:500; Cell Signaling) diluted in PBS containing 1.5% normal goat serum, overnight at 4 °C. Slides were washed in PBS before incubation with secondary antibodies (Alexa 488 goat anti rabbit; 1:400; Life Technologies) in PBS containing 1.5% normal goat serum for 1 h at room temperature. Finally, slides were washed in PBS and coverslipped using Vectashield mounting medium containing DAPI (Vector Laboratories). Images were acquired with an Olympus confocal microscope (FV1200, Olympus Life Science).

For the analysis of eGFP expression in Nestin-δ-HSV-TK mice, animals were trancardially perfused with 4% PF and the brains removed and post-fixed in 4% PF overnight at 4 °C. The brains were embedded in ascending concentration of glucose solutions (10, 20, 30% glucose in PBS) before embedding in OCT compound. Sections (40 μm thick) were cut on a cryostat and collected in antigen preservation solution containing 50% ethylene glycol and 0.25 M polyvinyl pyrrolidone (ThermoFisher Scientific, Waltham, MA) in PBS and stored at − 20 °C until ready to use. For immunofluorescence, free-floating sections were washed 3 times in PBS, blocked and permeabilized in 10% normal goat serum/0.3% Triton X-100/0.3 M Glycine in PBS 30 min and incubated with primary antibodies (chicken anti-GFP, 1:1000, Aves and mouse anti-NeuN, 1:400, Invitrogen) in 1.5% normal goat serum in PBS overnight at 4 °C for at least 18 h. After washing in PBS contain 0.3% Tween-20 (2 times, 10 min each), sections were incubated with Alexa-conjugated secondary antibodies (goat anti-chicken and goat anti-mouse, 1:400; Life Technologies) for 1 h at room temperature, washed and incubated with a DAPI solution (1:1000) for 5 min, rinsed in distilled water and mounted on microscope slides with FluorSave mounting medium (Calbiochem # 345789).

#### Small RNA deep sequencing and qRT-PCR confirmation analysis

Total RNA was isolated from NSCexo and MNexo using RNAeasy isolation kit (Qiagen, 3 separate preparations for each cell type) and processed for small RNA deep sequencing on an Illumina HiSeq1500 (Next Generation Sequencing Core at UTMB). The global quality of the RNA-seq dataset was tested by checking the reproducibility among technical replicates (Spearman R2 > 0.9) and for possible batch effects. Deep sequencing data was analyzed using the mirDeep2 miRNA analysis software suite. This program provides functions for trimming adapter sequences, mapping reads to known miRNAs, predicting novel miRNAs, and quantifying the expression levels of both known and novel miRNAs. Differential expression was analyzed using DESeq2 software.

Quantitative real time PCR with specific TaqMan probes (Applied Biosystems) was used to confirm the expression of miR-17, miR-322 and miR-485. Analysis of the published peer-review literature as well as in silico bioinformatics analysis using several prediction algorithms (TargetScan, GSEA, DESeq2) [56–58] was performed to identify specific miRNAs regulating synaptic function and plasticity.

#### Intracerebroventricular (ICV) injection of miRNA mimics and target engagement validation

Wild type mice were injected ICV with miRNA mimics (scrambled, miR-17, miR-322, miR-485) (ThermoFisher Scientific, Waltham, MA) dissolved in ACSF.

 Scrambled miRNA was delivered at 1 nmole per mouse, while 0.33 nmole of miR-17, miR-322 and miR-485 were mixed together and a final concentration of 1 nmole of miRNA per mouse was administered. Four animals per group were used. Twenty four hours after ICV injection, RNA was extracted from hippocampi of ICV injected mice using the RNeasy Mini Kit (Qiagen, Venlo, Netherlands) and cDNA was generated with the amfiRivert cDNA Synthesis kit (GenDEPOT, Barker, TX). Quantitative RT-PCR was performed using specific sense and antisense primers for known targets (STAT3, SYN5X, HIFa3) in a 20 μl reaction volume containing 10 μl of KAPA SYBR FAST qPCR Master mix (Kapa Biosystems), 0.5 μl of 10μmol/L primer stock, 1 μl cDNA, and 8 μl double-distilled H_2_O. Data were normalized to β-actin.

#### Aβo binding to exosomes in vitro

Aβo binding to exosomes was performed according to the procedure described by Yuyama et al, [59]. Briefly, PKH26-labelled exosomes were resuspended in PBS and plated onto chambered glass slides (Thermo Fisher Scientific, Whatman, MA) and allowed to sediment for 1 h at room temperature. Fluor 488-Aβo (1 μM) were added to the chamber and co-incubated for 5 h at 37 °C. After a wash with PBS to remove free Aβo, fluorescent images were taken using an Olympus Fluoview FV-10 confocal microscope.

### Statistical analysis

Data is expressed as mean+/− SEM. Analysis of variance (ANOVA) followed by multiple comparisons post-hoc tests were performed using GraphPad Prism software. Behavioral data was analyzed using either one-way ANOVA (Kruskal-Wallis post-hoc test) or repeated measures one or two-way ANOVA followed by a paired or unpaired t-test when significance was achieved. Post-hoc analysis was done using Mann-Whitney U or Wilcoxon matched pair test as appropriate for pair-wise comparison. Statistical significance was defined at *p* < 0.05.

## Results

### Isolation and characterization of exosomes

**Fig. 1:**
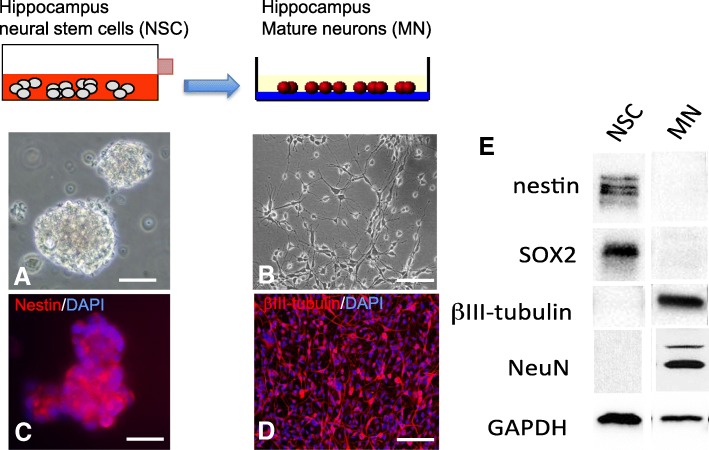
Characterization of hippocampus neural stem cells and mature neurons. Representative phase contrast images of adult rat hippocampus neural stem cells (NSC) maintained in culture as floating neurospheres (**a**) or differentiated into single-layer mature neurons (MN) (**b**) as confirmed by the expression of specific makers nestin (**c**) and βIII-tubulin (**d**), respectively. Scale bar = 50 μm. (E) Representative western blot of NSC and MN total protein lysate showing the expression of stem cell markers (nestin and sox2) and the lack of expression of neuronal (NeuN and βIII-tubulin) markers in the NSC, and lack of stem cell markers (nestin and sox2) and expression of neuronal markers (βIII-tubulin and NeuN) in MN. Please note that the western blot in (**e**) is a composite image of several blots shown here in aggregate for clarity and demonstrative purposes. Images are representative of over 20 independent experiments using hippocampus NSC between passages 6–10

**Fig. 2:**
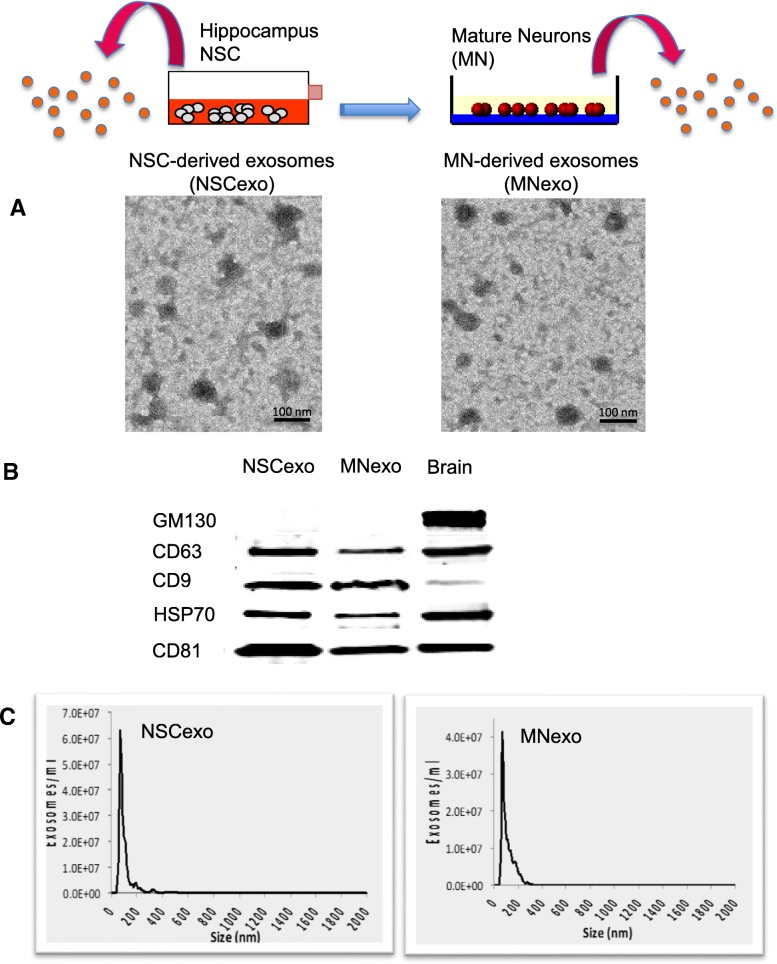
Characterization of exosomes isolated from the conditioned medium of neural stem cells (NSC-exo) and mature neurons (MN-exo). **a**) Representative high-resolution transmission electron microscopy image of exosomes secreted from NSC and from MN, derived from the differentiation of NSC, isolated using the ultra-centrifugation method (scale bar = 100 nm). **b**) Total protein lysates of isolated exosomes were analyzed by western blotting for the presence of exosome-specific proteins, CD63, CD9, HSP70, CD81, and the absence of the Golgi-associated protein GM130 as a control for cellular contamination. Total brain lysate was used as positive control. Please note that the western blot in (B) is a composite image of several blots shown here in aggregate for clarity and demonstrative purposes. Images are representative of over 20 independent experiments using hippocampus NSC between passages 6–10. **c**) Nanoparticle tracking analysis (NanoSight NS300, Malvern Panalytical, Malvern, UK) of exosomes isolated from NSC and MN shows a homogenous size distribution with an average single peak at 72.3 nm (NSC-exo) and 69.4 nm (MN-exo) (data is representative of 3 independent exosome preparations)

In this study, we used exosomes secreted from adult rat hippocampal neural stem cells (NSC) and from mature neurons (MN) obtained by differentiation of the NSC. Immunocytochemistry and western blot analysis confirmed the purity of the cultures by identifying the presence or absence of specific NSC (Sox2 and nestin) and neuronal (βIII-tubulin and NeuN) markers (Fig. 1). Exosomes were isolated from the cells’ conditioned culture media and characterized using electron microscopy, western blotting and single particle tracking analyses, according to the guidelines of the International Society of Extracellular Vesicles [60, 61]. High-resolution transmission electron micrographs confirmed that exosomes secreted by NSC (NSC-exo) and MN (MN-exo) exhibit rounded and double-membrane structures (Fig. 2a). The purity of the isolation was confirmed by analyzing by western blotting the presence of proteins that are known to be associated with exosomes (CD9, CD63, CD81 and HSP70) and the absence of the Golgi-associated protein GM130 (an important control for cellular contamination) (Fig. 2b). Lastly, single particle tracking analysis using the NanoSight system (Malvern Inc.) showed a bell-shaped curve size distribution profile, indicative of a physically homogeneous population, with an average single peak at 72.3 nm (NSC-exo) and 69.4 nm (MN-exo) (Fig. 2c).

### NSC-exo and MN-exo delivered intracerebroventricularly (ICV) are taken up by neurons

**Fig. 3:**
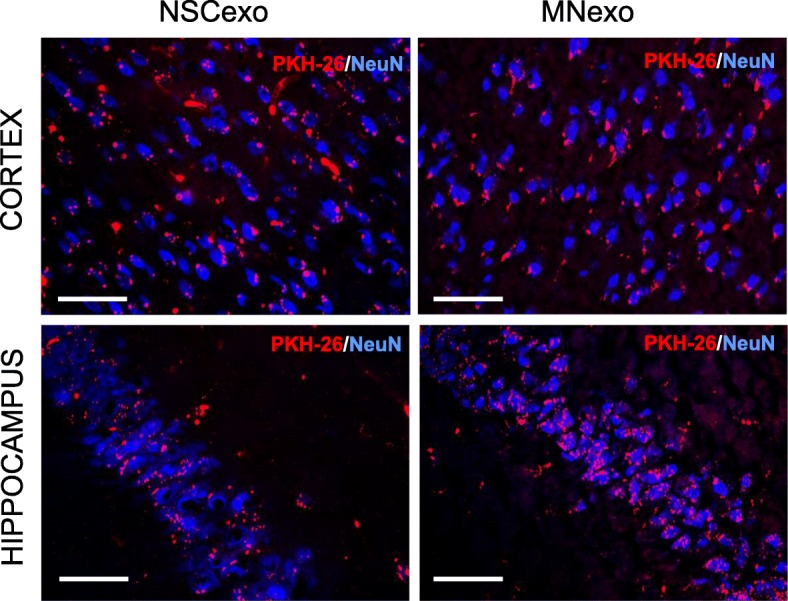
NSC-exo and MN-exo injected ICV diffuse in the brain parenchyma and are taken up by neuronal cells. Exosomes prepared from the conditioned medium of NSC (NSC-exo) and NSC-derived mature neurons (MN-exo) were labeled with PKH-26 and injected ICV in adult mice four hours before euthanasia. Representative confocal images of mouse brain cross sections showing numerous labeled exosomes (shown in red) in neuronal cells (identified by NeuN immunofluorescence: shown in blue) in the cortex and hippocampus. Images are representative of 3 independent experiments. Calibration bar = 50 μm

In order to determine the extent to which exogenously-administered exosomes secreted from NSC and MN enter the brain parenchyma and are taken up by neurons, NSC-exo and MN-exo were labeled with the membrane permeant cell tracker dye PKH-26 and injected ICV in adult wild type mice (3 × 10^9^ exosomes in 3 μl of sterile saline). The mice were euthanized 4 h later and the brains removed and processed for PKH-26 signal detection using confocal microscopy. Numerous PKH-26-labeled exosomes were seen in neuronal cells, identified by NeuN immunoreactivity, in the cortex and hippocampus (Fig. 3).

### NSC-exo prevent Aβ oligomer (Aβo)-induced memory deficits

**Fig. 4:**
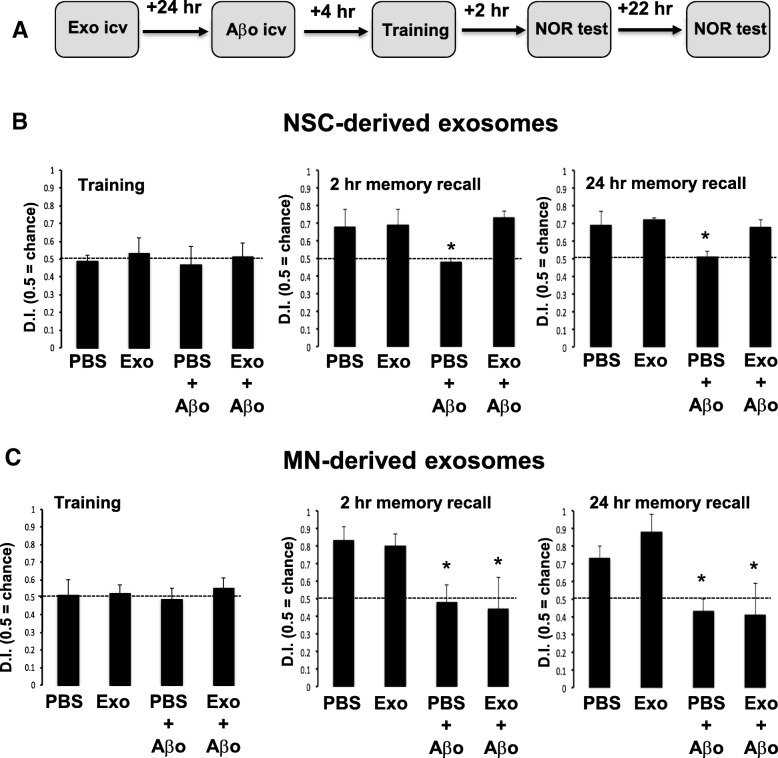
Aβo-induced memory deficit is rescued by NSC-exo but not by MN-exo. **a**) Schematic of the experimental design. Mice were injected icv with either NSC-exo (**b**) or MN-exo (**c**) 24 h prior to ICV injection of Aβ oligomers and testing in the novel object recognition (NOR) memory test. Aβ oligomers significantly impaired memory in mice pre-treated with vehicle (PBS) or with MN-exo, but not in mice pre-treated with NSC-exo *: not significant vs. chance and *p* < 0.05 vs. PBS-injected control mice (χ2 and ANOVA: F = 4.138 for 2 h recall test; F = 8.460 for 24 h recall test). *N* = 5 mice/group

In order to test whether exosomes secreted from NSC can protect from Aβo-induced memory impairment, wild type mice were injected ICV with NSC-exo, MN-exo or PBS 24 h prior to receiving ICV injections of Aβo (3 μL of a 95 μM stock) or PBS (3 μl). Four hours later, mice were subjected to the novel object recognition (NOR) training followed by memory recall tests at 2 h and 24 h thereafter (see treatment schematic in Fig. 4a). Animals in all groups equally explored the two identical objects during the training phase, indicating no effect of the various treatments on their natural exploratory behavior (Fig. 4b, c). Also, similar to the PBS treatment, ICV injection of MN-exo or NSC-exo alone did not affect the ability of the mice to discriminate between the familiar and the novel object at both 2 and 24 h memory recall testing times. However, as expected [50], mice treated with PBS or MN-exo that were further injected ICV with Aβo spent an equal amount of time exploring the familiar and the novel object at both the 2 h and 24 h memory recall tests, indicating impaired memory (Fig. 4c). On the other hand, animals that were treated ICV with NSC-exo prior to receiving Aβo spent significantly more time exploring the novel object at both times tested, revealing preserved memory function despite the administration of the toxic Aβo (Fig. 4b). Representative movement traces of the mice during the NOR testing are shown in Additional file 2: Figure S2.

### NSC-exo protect the hippocampus from Aβo-induced suppression of long-term potentiation (LTP)

**Fig. 5:**
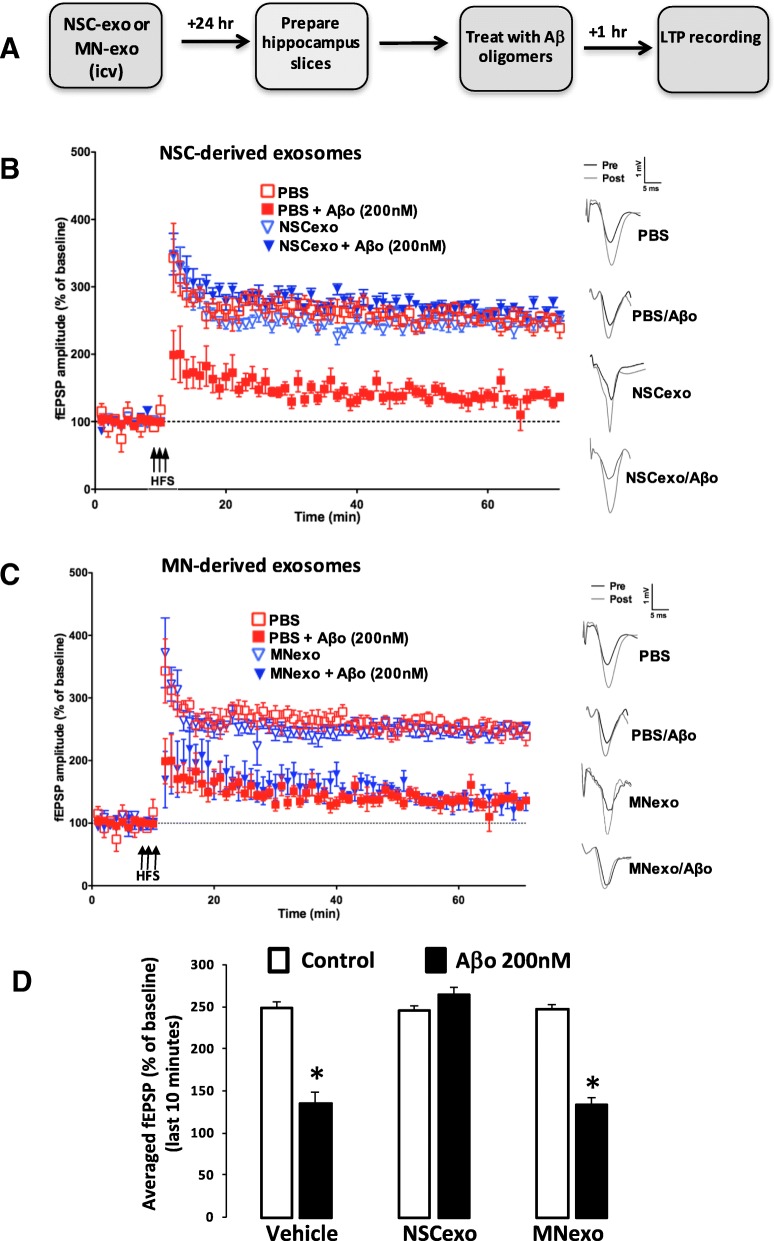
Aβo-induced suppression of long-term potentiation (LTP) in the hippocampus is abolished by ICV injection of NSC-exo but not by MN-exo. **a**) Schematic of the experimental design. NSC-exo, MN-exo or PBS (vehicle) were injected icv into adult mice 24 h before euthanasia. Schaffer collateral field recording of LTP (indicated as percent of baseline in the slope of fEPSPs) was performed on brain slices prepared from NSC-exo-treated mice (B) and MN-treated mice (**c**) in the presence of Aβ oligomers. Control mice were injected with PBS. Aβo abolished LTP in PBS treated mice and in MN-exo-treated mice but not in NSC-exo-treated mice. **d)** The fEPSP amplitude for the final 10 min (time points 50–60 min post high frequency stimulation) were averaged for each condition. Aβ oligomers significantly reduced LTP in brain slices from mice injected with vehicle or with MN-exo, but not in brain slices from mice treated with NSC-exo. *N* = 6 mice/group (2 slices per mouse). *p < 0.05 Student’s two-tailed T-test.

In order to investigate the possible cellular basis of the protective effect of NSC-exo on Aβ-induced memory deficits, we determined expression of LTP in hippocampal slices prepared from NSC-exo-injected mice and challenged with Aβ oligomers in a well-established protocol of Aβo-driven LTP suppression [45]. NSC-exo or MN-exo (3 × 10^9^ exosomes in 3 μl of sterile saline) were injected ICV in wild type mice 4 h or 24 h before euthanasia. Hippocampal slices were prepared and treated for one hour with either PBS or preformed Aβo (200 nM) (Fig. 5a and Additional file 3: Figure S3). After a stable baseline of 10 min, slices were subjected to high frequency stimulation (HFS) for one minute, and LTP was measured for an hour thereafter. We observed an increase in LTP (~ 250%) in slices from animals treated with PBS, MN-exo or NSC-exo, confirming that exosomes per se did not affect expression of LTP. Treatment of hippocampal slices with Aβo significantly reduced expression of LTP (~ 150%) in brain slices prepared from animals injected with either PBS or MN-exo (Fig. 5c), but not in slices from animals treated with NSC-exo (Fig. 5b). Quantitative assessment of LTP during the last 10 min of the recording (Fig. 5d) confirmed a statistically significant decrease in HFS-LTP induced by Aβo in hippocampus slices from PBS-treated and MN-exo-treated, but not in slices from NSC-exo-treated animals. This suggests that, consistent with their memory-protecting effects, NSC-exo reduce hippocampal synapses susceptibility to Aβo-driven LTP dysfunctions.

### NSC-derived exosomes promote hippocampal synaptic resistance to Aβo binding

**Fig. 6:**
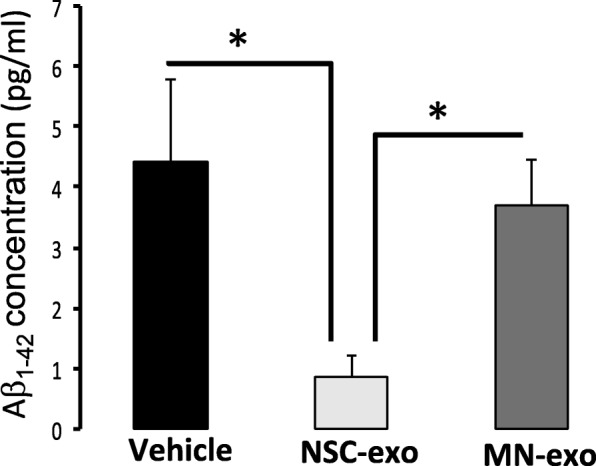
NSC-exo, but not MN-exo, reduce hippocampal synaptic vulnerability to Aβo binding. Fresh hippocampal brain slices were prepared from male wt mice and treated with NSC-exo or MN-exo for 4 h before being challenged with Aβ oligomers (2.5 μM for 30 min). Synaptosomes were then isolated from each brain slice and the amount of bound Aβ measured using a specific ELISA. *N* = 3 mice/group, 3 slices/mouse. *p < 0.05; Student’s two-tailed T-test

Given the observed protective effects of NSC-exo on Aβo-driven disruption of memory and LTP expression, we investigated whether exosomes secreted from hippocampal NSC can reduce synaptic vulnerability to Aβo binding. In these experiments, we elected to treat directly hippocampal slices to exclude possible confounding peripheral effects of exosomes. Freshly prepared hippocampal slices were maintained in ACSF and incubated with PBS, NSC-exo or MN-exo (3 × 10^9^ exosomes in 3 μl of sterile saline directly added to the ACSF) for 4 h, consistent with the earliest time resulting in robust uptake of exosomes by neurons (Fig. 3) and protection from Aβo-induced suppression of LTP (Additional file 3: Figure S3). At the end of the incubation, slices were washed 3 times with several volumes of fresh ACSF and further treated with Aβo (2.5 μM) for 30 min. ELISA was used to detect bound Aβ in synaptosomes isolated from hippocampal slices. As shown in Fig. 6, the amount of Aβo bound to hippocampal synaptosomes prepared from hippocampal slices treated with NSC-exo was significantly reduced as compared to the amount bound to synaptosomes from slices treated with PBS or MN-exo (*p* < 0.05 NSC-exo vs PBS and MN-exo).

**Fig. 7:**
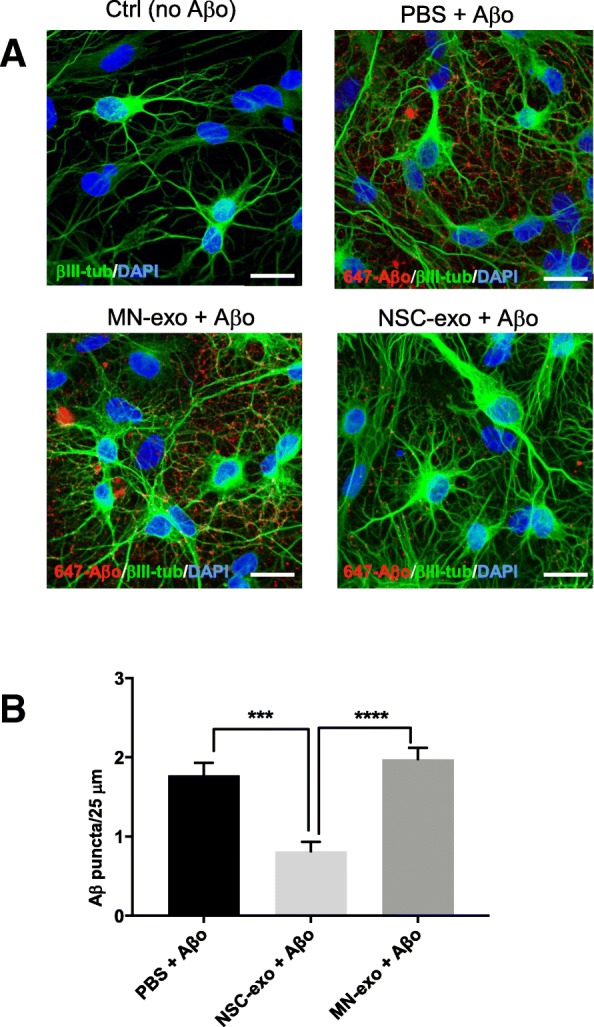
NSC-exo, but not MN-exo, reduce synaptic vulnerability to Aβo binding in hippocampus neurons in vitro. **a**) Representative confocal microscope images (40X w/3.2 zoom) showing association of Aβ oligomers (2.5 μm, 30 min; 647-Aβo, red) to hippocampal neurons (βIII-tubulin, green) generated by differentiation of adult rat hippocampus NSC. Nuclei are counterstained blue with DAPI. Calibration bar = 25 μm. **b**) Quantification of the number of 647-Aβο puncta shown in panel A (*N* = 12 images acquired from 4 independent experiments). ***p < 0.001; ****p < 0.0001 one-way ANOVA (F = 18.95) followed by Tukey’s multiple comparisons test

To further test whether NSC-exo-mediated reduction of Aβo binding at the synapse was due to a direct action of the exosomes on the neurons, cultures of mature hippocampus neurons, generated from the differentiation of NSC, were treated with NSC-exo or MN-exo for 24 h. After washing, Fluor 647-Aβo (2.5 μM) were added to the cultures for 30 min. After washing, the cells were fixed and stained with anti-βIII-tubulin antibody. Images taken with a confocal microscope revealed, as expected [62, 63], the presence of Aβo bound to the dendritic processes (Fig. 7a). Noticeably fewer Aβo were present in cultures pre-treated with NSC-exo, but not with MN-exo. Quantitative analysis performed on images from three independent experiments confirmed that NSC-exo treatment significantly reduced the amount of Aβo bound to the neuronal processes (Fig. 7b). Thus, these data suggest that decreased Aβo binding occurs in NSC-exo treated neurons.

**Fig. 8:**
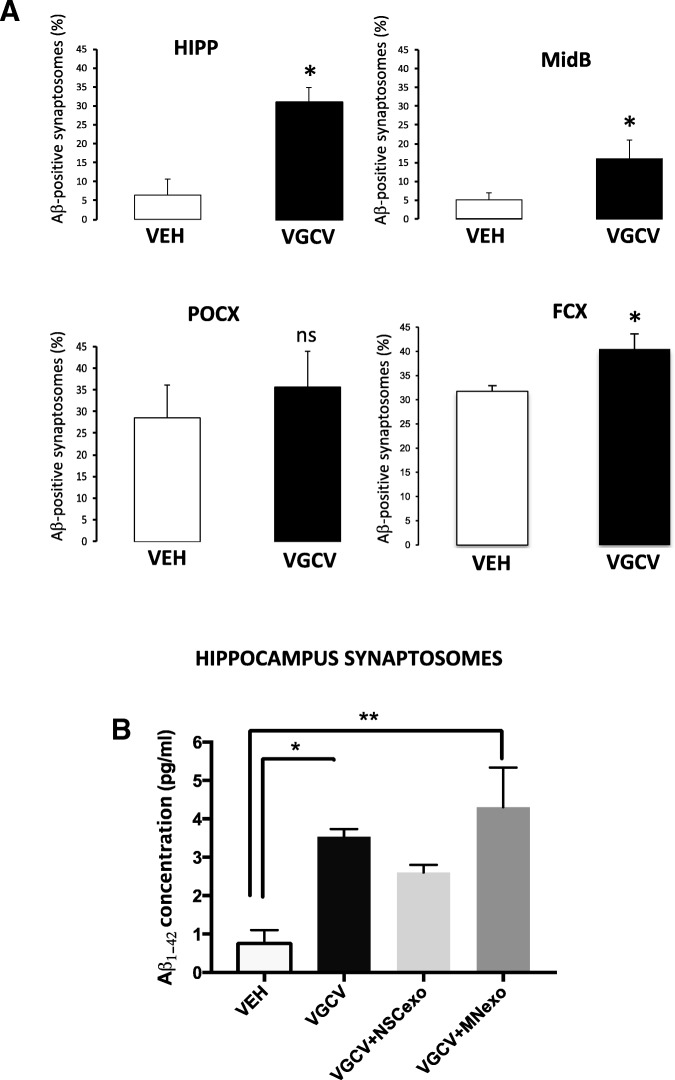
Hippocampal synaptic vulnerability to Aβo binding is increased in mice with ablated NSC and this increase is rescued by ICV injection of NSC-exo. **a**) Nestin-δ-HSV-Tk mice were fed vehicle or valganciclovir (VGCV) chow for 4 weeks. At the end of the 4 weeks, mice were euthanized and synaposomes were isolated from the hippocampus (HIPP), mid brain (MidB), frontal cortex (FCX) and parieto-occipital cortex (POCX) and challenged with Fluor 647-Aβ oligomers (2.5 μM) for 30 min. The percent of synaptosomes with bound Aβ was evaluated using flow cytometry. Mice treated with VGCV (with ablated NSC) showed a significant increase in the percent of Aβ positive synaptosomes as compared to vehicle-treated mice (with intact NSC) in the hippocampus, midbrain and frontal cortex. No statistically significant differences were noted in the parieto-occipital cortex. *N* = 6 mice/group. *p < 0.05 Student’s two-tailed T-test. **b**) Nestin-δ-HSV-Tk mice were fed vehicle or valganciclovir (VGCV) chow for 4 weeks. At the end of the 4 weeks, fresh hippocampal brain slices were prepared and treated with NSC-exo or MN-exo for 4 h before being challenged with Aβ oligomers (2.5 μM for 30 min). ELISA was used to measure the amount of Aβ bound to synaptosomes. N = 3 mice/group. *p < 0.05; **p < 0.01 one-way ANOVA (F = 7.276) followed by Tukey’s multiple comparisons test

Lastly, to explore whether endogenous NSC affected synaptic sensitivity to Aβo, we temporally ablated NSC from the hippocampus of Nestin-δ-HSV-TK-eGFP transgenic mice [42]. This transgenic line contains a modified version of the herpes simplex virus thymidine kinase (TK), as well as an enhanced green fluorescent protein (GFP), driven by the nestin promoter and its second intron regulatory element. Administration of valganciclovir in mouse chow, which is specifically phosphorylated by Nestin-δ-HSV-TK, kills dividing nestin expressing cells in these mice by acting as a toxic thymidine analog. GFP-expressing nestin positive cells in the subgranular layer of the hippocampus and in the subventricular zone of the lateral ventricle (the two main neurogenic zones in the adult rodent brain) were ablated in Nestin-δ-HSV-TK-eGFP mice treated with valganciclovir as compared to vehicle-treated mice, confirming the validity of the model (Additional file 4: Figure S4). We then assessed Aβo binding in vitro, using flow cytometry, in synaptosomes isolated from the hippocampus (HIPP), midbrain (MidB), parieto-occipital cortex (POCX) and frontal cortex (FCX) of Nestin-δ-HSV-TK-eGFP mice treated for 4 weeks with valganciclovir (VGCV) or vehicle. As shown in Fig. 8a, animals lacking resident NSC displayed a significant increase of Aβo binding in hippocampus, midbrain and frontal cortex as compared to vehicle-treated mice with normal NSC numbers. On the other hand, Aβo binding to parietal cortex synaptosomes was also noticeably increased upon transgenic suppression of NSC but did not reach statistical significance.

In order to determine whether such an increase of synaptic Aβo binding occurred as a function of reduced levels of NSC-derived exosomes, hippocampus slices were prepared from vehicle-treated and VGCV-treated Nestin-δ-HSV-TK-eGFP mice with ablated NSC and treated with NSC-exo or MN-exo for 4 h. After washing, the slices were further treated with Aβo (2.5 μM) for 30 min and bound Aβ was detected by ELISA in isolated synaptosomes. As shown in Fig. 8b, and consistent with the results shown in Fig. 8a, synaptosomes from VGCV-treated Nestin-δ-HSV-TK-eGFP mice showed a significantly increased binding of Aβo as compared to synaptosomes from vehicle-treated Nestin-δ-HSV-TK-eGFP mice with normal NSC numbers. Such increased synaptic vulnerability was corrected by treating the slices with NSC-exo but not with MN-exo prior to exposure to Aβo, suggesting that NSC-exo are sufficient to compensate for the loss of endogenous NSC in terms of synaptic sensitivity to Aβo.

### Phosphorylated (active) CaMKII is increased in the synapses of mice treated ICV with NSC-exo

**Fig. 9:**
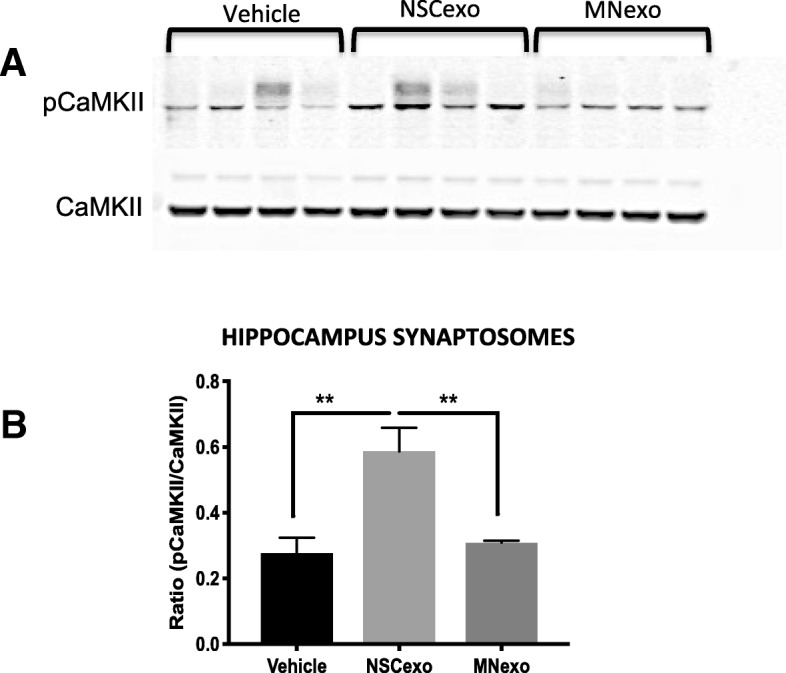
CaMKII phosphorylation is increased in the synapses of mice treated ICV with NSC-exo. NSC-exo, MN-exo or PBS (vehicle) were injected ICV into adult wild type mice 24 h before euthanasia. Synaptosomes were isolated from the hippocampus and analyzed by western blotting for the expression of total and phosphorylated Ca^2+^/calmodulin-dependent kinase II (CaMKII) (**a**). Band intensities were quantified using ImageJ software and normalized to β-actin (**b**). *N* = 4 mice/group. ANOVA (F = 11.83) followed by Tukey’s multiple comparisons test

In order to determine whether the protective effect of NSC-exo could be in part mediated by changes in the expression and/or activation of proteins relevant to Aβo-induced toxicity, we isolated hippocampal synaptosomes 24 h after ICV injection of PBS (vehicle), NSC-exo or MN-exo and analyzed the total protein lysates by western blotting. We found that phosphorylation of Ca^2+^/calmodulin-dependent kinase II (CaMKII) was significantly increased in the synaptosomes of mice treated with NSC-exo as compared to MN-exo and vehicle (Fig.9). On the other hand, NSC-exo treatment did not change the expression of AMPA (GluR1, GluR2) and NMDA (NR1, NR2) glutamate receptor subunits at the synaptosome (Additional file 5: Figure S5).

### Specific miRNAs in NSC-exo reduce Aβo binding to the synapse and protect the hippocampus from Aβo-induced suppression of LTP

**Fig. 10:**
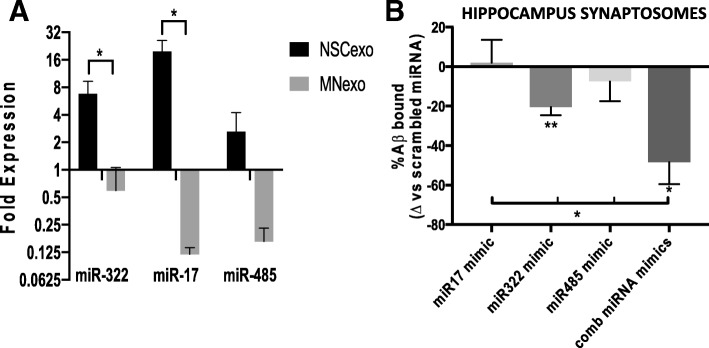
ICV injection of mimics of miRNAs enriched in NSC-exo reduces hippocampal synaptic vulnerability to Aβo binding. **a**) Relative levels of expression of miR-322, miR-17 and miR-485 in NSC-exo as compared to MN-exo as determined by small RNA deep sequencing. *N* = 3 independent NSC-exo or MN-exo preparations each assayed in 3 independent deep sequencing analyses. * p < 0.05 Student’s T-test. **b**) Mimics of miRNAs (alone or in combination) (1 nmole if injected alone or 0.33 nmoles each if injected in combination) or scrambled miRNA as control were injected ICV 24 h before sacrifice. Hippocampus synaptosomes were prepared and challenged with preformed Aβo (200 nM). After washing, the amount of Aβ in the synaptosomes was quantified by ELISA. *p < 0.05; **P < 0.01 vs. scrambled miRNA. N = 3–4 mice/group. *p < 0.05; *p* < 0.01 ANOVA (F = 4.796) followed by unpaired T-test multiple comparison analysis.

In order to determine whether select miRNAs uniquely present in NSC-exo could mediate their protective action on synapses of target neurons, we performed small RNA Deep sequencing analysis and identified a set of unique miRNAs enriched in NSC-exo, as compared to MN-exo, involved in regulation of synaptic function and plasticity (Additional file 6: Figure S6). Of these unique NSC-exo miRNAs, we selected the most abundant ones (miR-322 and miR-17) along with miR-485 (Fig. 10a). Although miR-485 was enriched in NSC-exo to a lesser extent than miR-322 and miR-17, we included it in our study because in our previous work we have identified it as an upstream regulator of proteomics changes observed at the synapses of human hippocampus autoptic samples from resilient NDAN individuals [20]. Mimics of these miRNAs were injected ICV into wild type mice 24 h before injection of Aβo. Mice were euthanized 24 h later. We found that mimics of miR-322 and miR-485 significantly reduced binding of Aβo to hippocampal synaptosomes as compared to scrambled miRNA control, while miR-17 had no effect (Fig. 10b). Notably, we found that when the mice were injected ICV with the combination of all three miRNAs mimics (maintaining the same overall amount of injected RNA), there was a greater decrease of Aβo association with the synapse (Fig. 10b), pointing to a synergistic effect of the mimics.

**Fig. 11:**
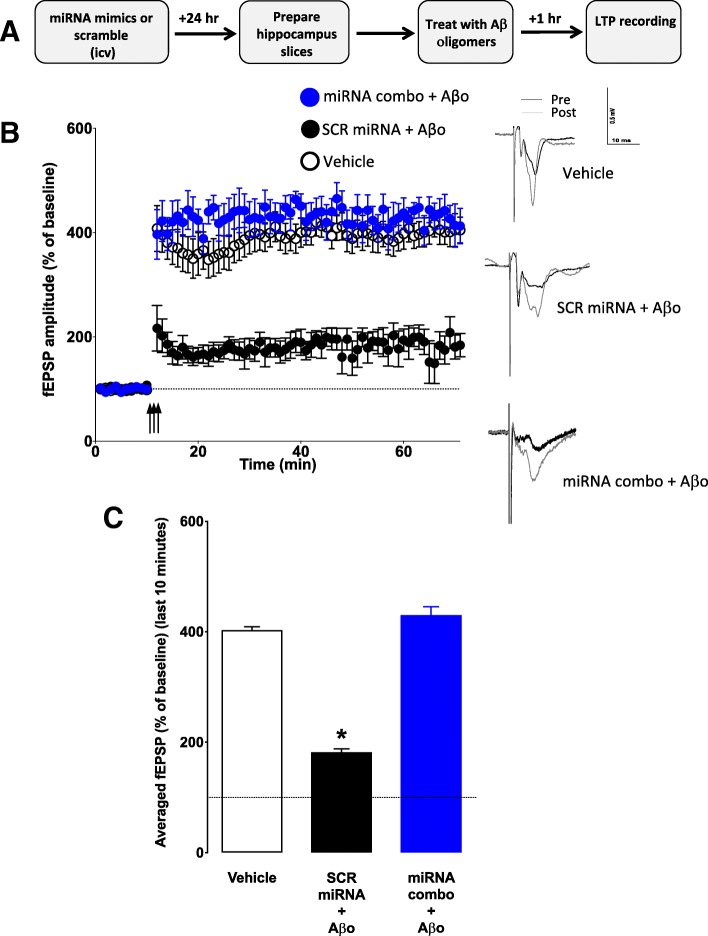
Aβo-induced suppression of LTP in the hippocampus is abolished by mimics of miRNAs enriched in NSC-exo. **a**) Schematic of the experimental design. MicroRNAs (scrambled or mimics of miR-322, miR17 and miR-485) or PBS (vehicle) were injected ICV into adult mice 24 h before euthanasia. Schaffer collateral field recording of LTP (indicated as percent of baseline in the slope of fEPSPs) was performed on brain slices in the presence of Aβ oligomers. Control mice were injected with PBS (vehicle). Aβo abolished LTP in PBS treated mice and in scrambled-treated mice but not in miRNAs combo-treated mice (**b**). **c**) The fEPSP amplitude for the final 10 min (time points 50–60 min post high frequency stimulation) were averaged for each condition. Aβ oligomers significantly reduced LTP in brain slices from mice injected with vehicle or with scrambled miRNA, but not in brain slices from mice treated with combo miRNAs mimics. *N* = 6 mice/group (2 slices per mouse). *p < 0.05 Student’s two-tailed T-test

In order to test whether reduced Aβo binding resulted in improved synaptic function, we injected ICV a combination of mimics for the three miRNAs (miR-485, miR-17 and miR-322), or the same amount of scrambled RNA, 24 h before euthanasia. Hippocampal slices were prepared and treated for one hour with either PBS or preformed Aβo (200 nM) (Fig. 11a). After a stable baseline of 10 min, slices were subjected to high frequency stimulation (HFS) for one minute, and LTP was measured for an hour thereafter. We found that Aβo-driven reduction of LTP was absent in mice treated with miRNA mimics (Fig. 11b-c).

## Discussion

We report here that in mice, exosomes derived from cultured NSC render neuronal synapses less vulnerable to the binding of toxic Aβo, protect synapses from Aβo-induced suppression of LTP expression and prevent memory deficits driven by ICV injection of Aβo. On the other hand, exosomes derived from MN obtained from differentiated NSC were not effective. Furthermore, we show that abolishing NSC in the hippocampus DG of inducible Nestin-δ-HSV-TK-eGFP tg mice increases synaptic vulnerability to Aβo and that this phenomenon is reversed by NSC-derived exosomes. Taken together, these data suggest the existence of a previously unappreciated mechanism whereby endogenous NSC modulate synaptic sensitivity to the dysfunctional binding of Aβo through local signaling via released exosomes.

Neurogenesis in the adult brain is known to support synaptic and cognitive function and recent reports from our group have demonstrated a direct correlation between the number of NSC in the hippocampus DG and preserved cognitive function in non-demented individuals with AD neuropathology (NDAN), suggestive of a protective role of hippocampal NSC [27]. Although this protective effect could be attributed to two main functions of NSC, endocrine secretion and generation of neurons, we have shown that the number of mature neurons in the hippocampus DG of NDAN individuals does not correlate with their preserved cognitive competency [27]. This suggests that the association between increased number of NSC and cognitive resilience in the face of AD pathology is likely mediated by an endocrine secretion mechanism rather than by the NSC ability to generate new neurons. An important endocrine secretion of NSC is constituted by exosomes, intercellular messengers implicated in the transfer of mRNA, miRNAs, proteins and lipids between cells [64]. Here, we show that exosomes secreted from NSC of the hippocampus DG render synapses resistant to Aβο binding and prevents related dysfunctions (suppression of LTP expression and memory recall deficits). Most importantly, our data show that this mechanism is specific to NSC because exosomes secreted by MN were unable to afford the synapses with a similar protection against Aβo. Using exosomes from MN derived from differentiation of NSC, as we did here, is an important strategy because comparing the functional effects of exosomes specifically associated with either a “stem” state or a mature neuronal phenotype of the same cell lineage provides a rigorous scientific control in support of our results, excluding the possibility of non-specific effects of the exosome vesicles such as scavenging of exogenously-added Aβο [65, 66]. Moreover, we did not find a significant association between Aβo and exosomes derived from NSC or MN (Additional file 7: Figure S7), thus further supporting our hypothesis that NSC-exo protect against Aβo-mediated toxicity by inducing changes in the recipient neurons. Indeed, we found that phosphorylation (activation) of CaMKII (a critical enzyme for activity-dependent synaptic remodeling associated with cognitive function) [67, 68] is significantly increased at the synapse after ICV treatment with NSC-exo. On the other hand, levels of NMDA and AMPA receptor subunits where not changed at the synaptosomes, indicating that changes in the levels of these receptors known to dock Aβo [69, 70] may not be a key factor in mediating synaptic resilience to Aβo afforded by NSC-exo. Rather, more global changes overall resulting in better synaptic function (as reflected by increased pCaMKII) may play a role.

While our data show that exosomes are taken up by neurons and are capable of reducing Aβo binding when administered to neuronal cultures, we cannot exclude that they can be taken up by glial cells when injected ICV. Although further studies are necessary to determine whether this is indeed the case and the functional significance of such event, our in vitro data in neuronal cultures clearly demonstrate that uptake of NSC-exo by neurons is sufficient to induce synaptic resistance to Aβo binding.

Binding of Aβo at the synapses is well-documented to result in disrupted synaptic function, ultimately leading to dendritic spine retraction and cognitive impairments [25, 62]. Our data show that both synaptic plasticity (LTP) and memory function (NOR test) are protected against Aβo-induced deficits in mice treated ICV with NSC-exo (but not MN-exo) prior to challenging with Aβo. Furthermore, we show that ablation of NSC in an inducible transgenic mouse model leads to increased synaptic vulnerability to Aβo that can be over-ridden by exogenous administration of NSC-exo but not MN-exo. Collectively these results indicate that exosomes specifically released by resident NSC play a key role in promoting or maintaining synaptic resilience to the damaging impact of Aβo that ultimately results in memory deficits. They further suggest the intriguing possibility that a decrease in the number of NSC in the hippocampus DG, such as that occurring during aging [71–73], could lead to increased synaptic vulnerability to the toxic binding of amyloid oligomers, possibly because of reduced NSC-exo signal to neurons. On these bases, it is not unreasonable to envision such a mechanism being one factor linking aging to increased risk of AD [74], although further studies are needed to ultimately confirm this possibility. Furthermore, exosomes released by affected neurons have been recently investigated in AD as a possible vehicle of propagation of pathological misfolded proteins, including tau [75, 76]. Our studies are not in contrast with this view. Rather, we introduce a complementary new concept specifically centered on NSC-secreted exosomes that, rather than being an alternative, provides an additional layer to the still poorly characterized complexity of extracellular vesicles in the CNS and their involvement in AD onset/progression.

MiRNAs are very abundant in exosomes and, in addition to proteins and lipids, represent their main bioactive cargo [77, 78]. Indeed, compelling evidence indicates that many of the effects elicited by exosomes (including NSC-exo) can be ascribed to the action of their specific miRNA cargoes, including modulation of aging [32], cognition and synaptic function [38–40] and neuroprotection [41]. Importantly, miRNAs have been shown to regulate transcriptional modulation of key synaptic proteins within the synaptic compartment itself [79, 80]. Furthermore, our own previous findings show that resilient hippocampal synapses in NDAN individuals with high numbers of NSCs [27] have a unique proteomic signature [20], suggesting that selective changes in synaptic protein expression (consistent with upstream regulation by specific miRNAs, as revealed by IPA analysis of our proteomic data) mark synaptic resistance to toxic Aβ and tau oligomers in these humans. We found that, as compared to MN-exo, NSC-exo contain a unique set of miRNAs that are known to modulate the expression of proteins involved in synaptic function. Most importantly, we also found that when injected ICV in mice, mimics of such unique NSC-exo derived miRNAs render synapses physically resistant to the detrimental binding of Aβo and functionally resilient to their disruptive action. These data support the notion that selected miRNAs uniquely present in NSC-exo could mediate their protective action on synapses of target neurons and further suggests the exciting possibility of new drug discovery to promote cognitive resilience in AD.

## Conclusions

In conclusion, our data point to NSC-secreted exosomes and their miRNAs cargo as one of the critical elements constructing the yet poorly understood neurobiological bases of the complex relationship linking brain reserve to cognitive resilience and resistance to AD neuropathology. Future pursuing of this novel concept will hopefully open the door to new therapeutic strategies for AD centered on NSC-secreted exosomes and their bioactive cargoes. Indeed, the effect of NSC-secreted exosomes in increasing synaptic resistance to amyloid oligomers has never been documented before and supports the development of a novel and attractive therapeutic strategy aimed at using NSC-secreted exosomes to delay the onset and/or mitigate the severity of Aβο-dependent synaptic and cognitive dysfunctions associated with age-dependent decrease of neurogenesis.

## Additional files

Additional file 1: **Figure S1.** Characterization of isolated synaptosomes. A) Representative high-resolution transmission electron microscopy images showing the typical morphology of synaptosomes with readily identifiable preserved synaptic structures (white arrows). B) Flow cytometry was used to count synaptosomes. Reference standard size beads (EMD Millipore) were used to gate particle sizes up to 5.6 μm for the analysis in order to exclude larger cellular debris. (PPTX 2676 kb)

Additional file 2: **Figure S2.** Representative movement traces of mice during the NOR testing Mice treated with NSC-exo 24 h before Aβo ICV injection spent more time exploring the novel object in a manner similar to that of control mice without Aβo (PBS or exo). On the other hand, mice treated with MN-exo before Aβo ICV injection spent the same amount of time exploring the familiar and the novel objects (indicative of impaired memory). (PPTX 889 kb)

Additional file 3: **Figure S3**. Aβo-induced suppression of LTP in the hippocampus is abolished by NSC-exo injected ICV four hours earlier. A) Schematic of the experimental design. NSC-exo, MN-exo or PBS (vehicle) were injected ICV into adult mice 4 h before euthanasia. Schaffer collateral field recording of LTP (indicated as percent of baseline in the slope of fEPSPs) was performed on brain slices prepared from NSC-exo-treated mice (B) and MN-treated mice (C) in the presence of Aβ oligomers. Control mice were injected with PBS. Aβo abolished LTP in PBS treated mice and in MN-exo-treated mice but not in NSC-exo-treated mice. D) The fEPSP amplitude for the final 10 min (time points 50–60 min post high frequency stimulation) were averaged for each condition. Aβ oligomers significantly reduced LTP in brain slices from mice injected with vehicle or with MN-exo, but not in brain slices from mice treated with NSC-exo. *N* = 6 mice/group (2 slices per mouse). *p < 0.05 two-tailed T-test. (PPTX 433 kb)

Additional file 4: **Figure S4.** Depletion of hippocampal neural stem cells following treatment of Nestin-δ-HSV-TK mice with valganciclovir. A) Construct scheme for Nestin-δ-HSV-TK transgenic mice. (B-G) Representative images of Nestin-δ-HSV-TK mice brain coronal sections showing the hippocampus dentate gyrus (B-E) and the subventricular zone (SVZ) of the lateral ventricle (F-G) stained with an antibody against green fluorescent protein (GFP, green) and neuronal nuclei (NeuN, red). GFP^+^ neural stem cells in the hippocampus dentate gyrus and SVZ are ablated after 4 weeks of Valganciclovir (VGCV) treatment (C, E, G) as compared to mice treated with vehicle (B, D, F). Calibration bar = 100 μm. (PPTX 1270 kb)

Additional file 5: **Figure S5.** Expression of NMDA and AMPA glutamate receptors in hippocampal synaptosomes. Total protein lysates of synaptosomes isolated from the hippocampus of mice injected ICV with PBS (vehicle), NSC-exo or MN-exo were analyzed by western blotting for the expression of total and phosphorylated glutamate AMPA (GluR1 and GluR2) and NMDA (NR1 and NR2) receptors (A). Band intensities were quantified using ImageJ software and normalized to β-actin. *N* = 3. *p < 0.05 Unpaired T-test. (PPTX 471 kb)

Additional file 6: **Figure S6.** Small RNA deep sequencing comparing RNA content in NSC-exo and MN-exo reveals that NSC-exo express a set of unique miRNAs involved in regulation of synaptic function and plasticity. A) Secreted exosomal miRNAs enriched in NSC-exo as compared to MN-exo. B) KEGG pathway analysis (*P* < 0.05) revealed potential target genes of these miRNAs enriched in pathways regulating synaptic function and plasticity. Each bar in blue indicates the number of miRNAs involved in the relevant pathway. The number of regulated genes involved in each pathway is indicated in parenthesis. Data is from 3 separate preparations from each cell type and 3 technical replicates. C) Mimics of miRNAs were injected ICV 24 h before sacrifice. The efficiency of the delivered mimics was confirmed by measuring levels of specific mRNAs regulated by the selected miRNAs, using RT-PCR. **P < 0.01; ***P < 0.001; ***P < 0.0001 vs. scrambled miRNA (T-test). *N* = 4 mice/group. (PPTX 230 kb)

Additional file 7: **Figure S7.** Aβo don’t associate with NSC-exo and MN-exo. Representative confocal images of PKH26-labelled exosomes (red) after 5 h incubation with fluorescent Aβ oligomers (Fluor 488-Aβo, 1 μM, green). No association of Aβo with exosomes is noted. Calibration bar is 10 μM. (PPTX 383 kb)

## Abbreviations

ACSF: Artificial cerebrospinal fluid
AD: Alzheimer’s disease
Aβo: Amyloid β oligomers
DG: Dentate gyrus
ELISA: Enzyme-linked immunosorbent assay
EM: Electron microscopy
Exo: Exosomes
GFP: Green fluorescent protein
ICV: Intracerebroventricular injection
LTP: Long-term potentiation
miRNA: MicroRNA
MN: Mature neurons
NDAN: Non-demented individuals with AD neuropathology
NOR: Novel object recognition test
NSC: Neural stem cells
PBS: Phosphate buffered saline
RIPA: Radioimmunoprecipitation assay
Tg: Transgenic
WB: Western blotting
Wt: Wild-type
