# Supplementary figures and images for: Hippocampal stem cells promotes synaptic resistance to the dysfunctional impact of amyloid beta oligomers via secreted exosomes
Source: Mol Neurodegener. 2019 Jun 14;14:25. doi: 10.1186/s13024-019-0322-8 (PMC6570890; doi:10.1186/s13024-019-0322-8)

## Slide 1
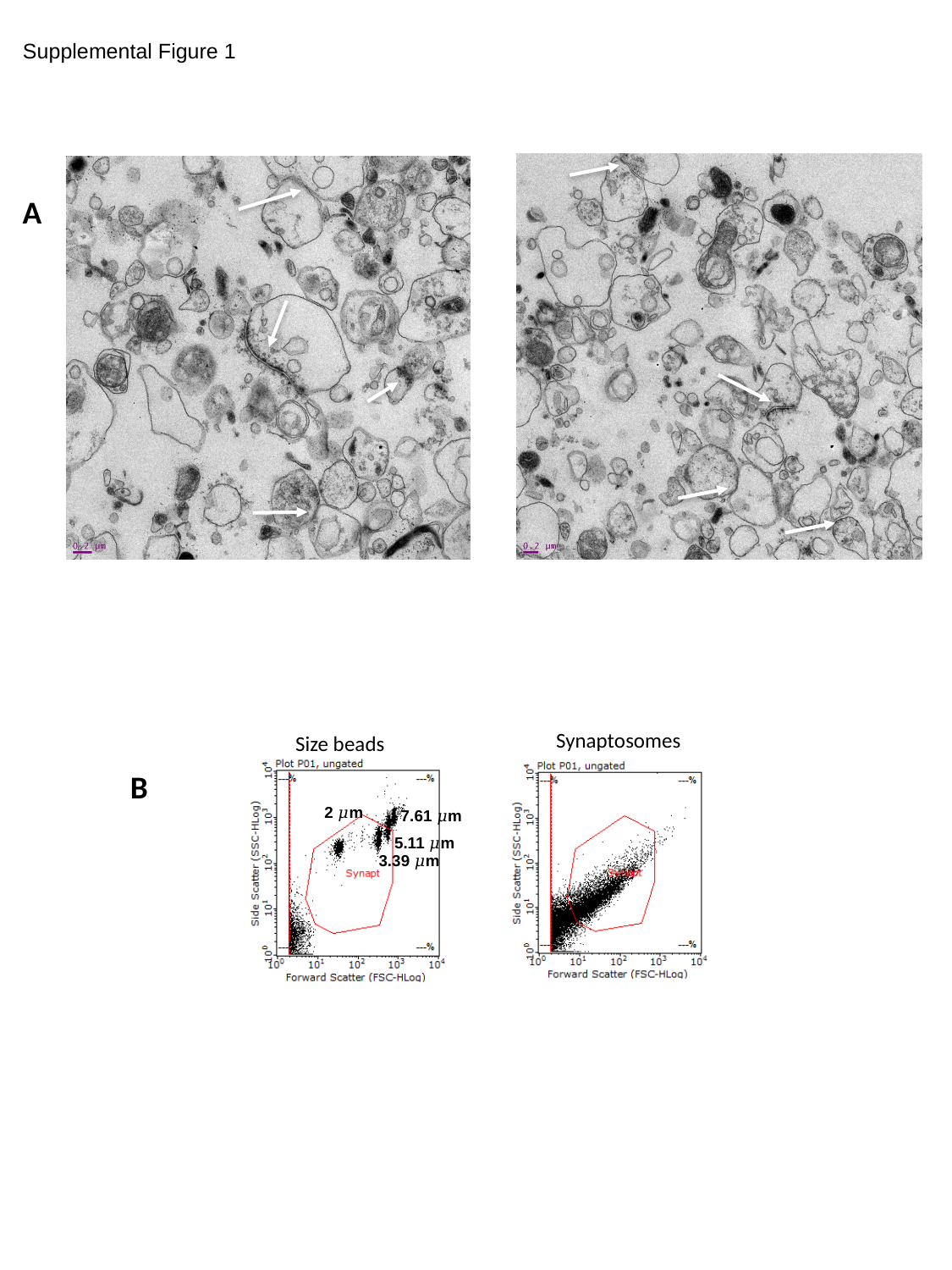

Supplemental Figure 1
A
Synaptosomes
Size beads
2 𝜇m
7.61 𝜇m
5.11 𝜇m
3.39 𝜇m
B

Supplement: Supplementary file 1 — Figure S1. Characterization of isolated synaptosomes. A) Representative high-resolution transmission electron microscopy images showing the typical morphology of synaptosomes with readily identifiable preserved synaptic structures (white arrows). B) Flow cytometry was used to count synaptosomes. Reference standard size beads (EMD Millipore) were used to gate particle sizes up to 5.6 μm for the analysis in order to exclude larger cellular debris. (PPTX 2676 kb) [file 13024_2019_322_MOESM1_ESM.pptx]

## Slide 1
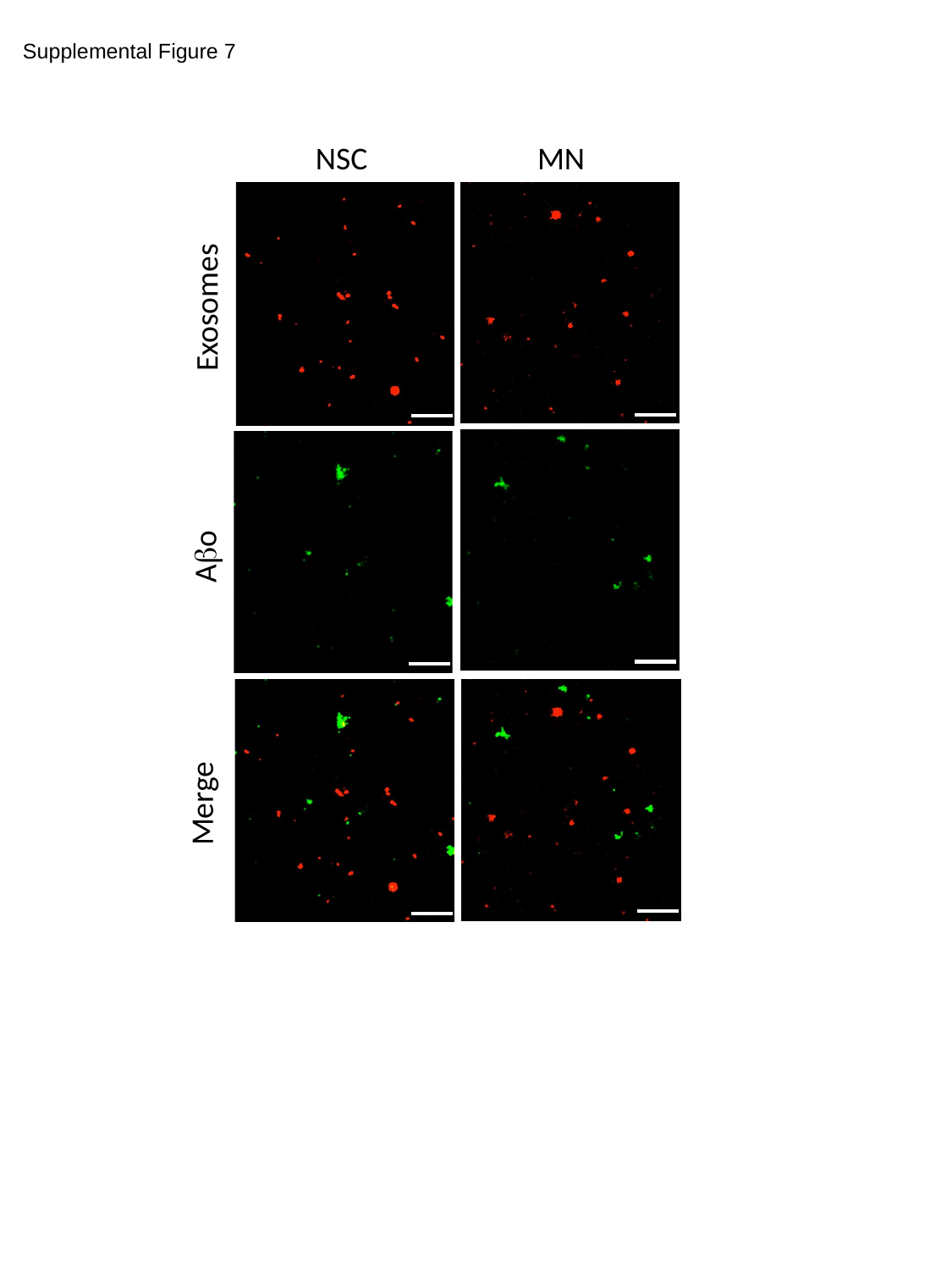

Supplemental Figure 7
NSC
MN
Exosomes
Abo
Merge

Supplement: Supplementary file 7 — Figure S7. Aβo don’t associate with NSC-exo and MN-exo. Representative confocal images of PKH26-labelled exosomes (red) after 5 h incubation with fluorescent Aβ oligomers (Fluor 488-Aβo, 1 μM, green). No association of Aβo with exosomes is noted. Calibration bar is 10 μM. (PPTX 383 kb) [file 13024_2019_322_MOESM7_ESM.pptx]
