# Supplementary material for: Hippocampal stem cells promotes synaptic resistance to the dysfunctional impact of amyloid beta oligomers via secreted exosomes
Source: Mol Neurodegener. 2019 Jun 14;14:25. doi: 10.1186/s13024-019-0322-8 (PMC6570890; doi:10.1186/s13024-019-0322-8)
Supplement: Supplementary file 2 — Figure S2. Representative movement traces of mice during the NOR testing Mice treated with NSC-exo 24 h before Aβo ICV injection spent more time exploring the novel object in a manner similar to that of control mice without Aβo (PBS or exo). On the other hand, mice treated with MN-exo before Aβo ICV injection spent the same amount of time exploring the familiar and the novel objects (indicative of impaired memory). (PPTX 889 kb) [file 13024_2019_322_MOESM2_ESM.pptx]

## Slide 1
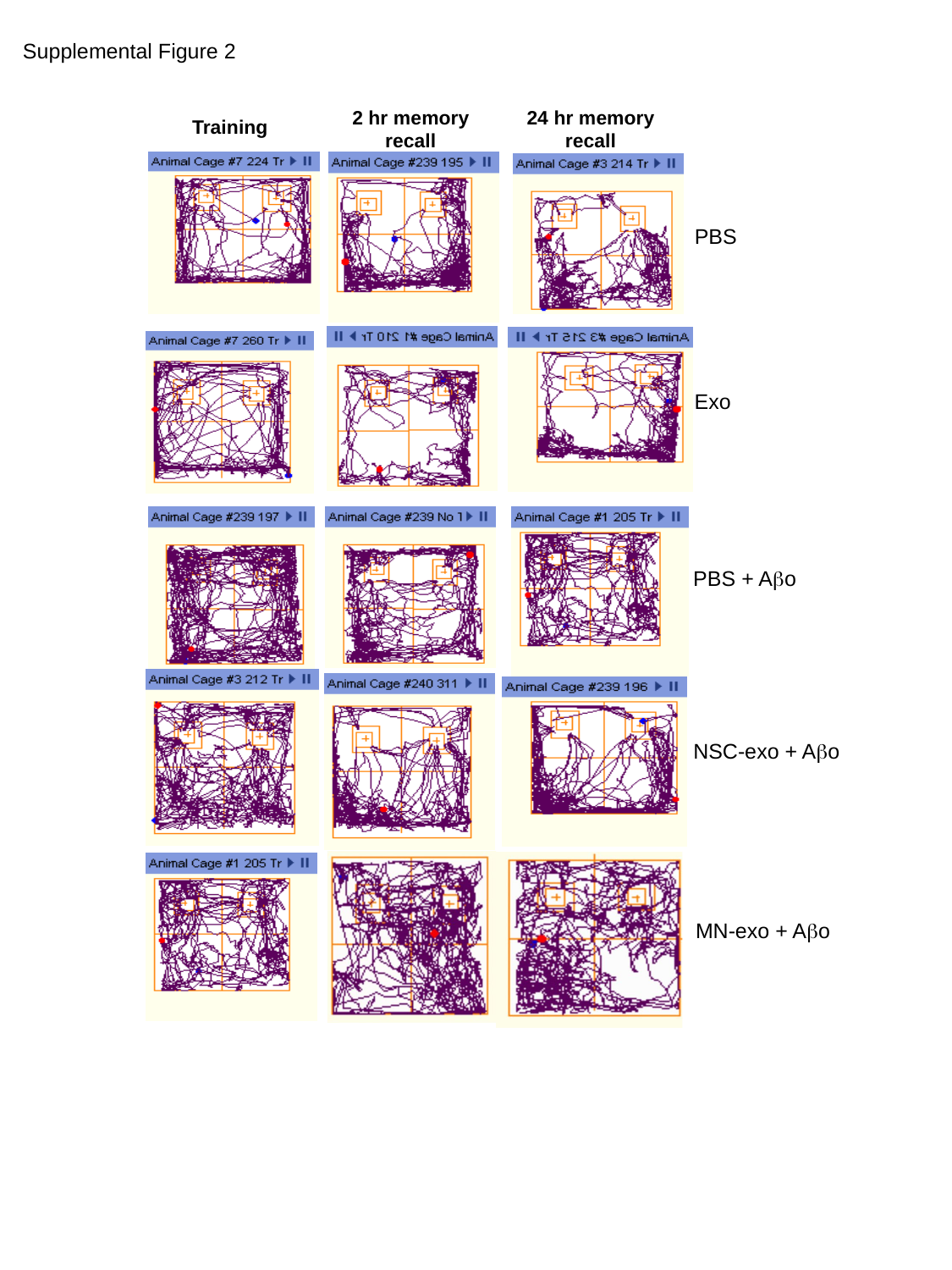

Supplemental Figure 2
24 hr memory recall
2 hr memory recall
Training
PBS
Exo
PBS + Abo
NSC-exo + Abo
MN-exo + Abo
