# Supplementary material for: Hippocampal stem cells promotes synaptic resistance to the dysfunctional impact of amyloid beta oligomers via secreted exosomes
Source: Mol Neurodegener. 2019 Jun 14;14:25. doi: 10.1186/s13024-019-0322-8 (PMC6570890; doi:10.1186/s13024-019-0322-8)
Supplement: Supplementary file 3 — Figure S3. Aβo-induced suppression of LTP in the hippocampus is abolished by NSC-exo injected ICV four hours earlier. A) Schematic of the experimental design. NSC-exo, MN-exo or PBS (vehicle) were injected ICV into adult mice 4 h before euthanasia. Schaffer collateral field recording of LTP (indicated as percent of baseline in the slope of fEPSPs) was performed on brain slices prepared from NSC-exo-treated mice (B) and MN-treated mice (C) in the presence of Aβ oligomers. Control mice were injected with PBS. Aβo abolished LTP in PBS treated mice and in MN-exo-treated mice but not in NSC-exo-treated mice. D) The fEPSP amplitude for the final 10 min (time points 50–60 min post high frequency stimulation) were averaged for each condition. Aβ oligomers significantly reduced LTP in brain slices from mice injected with vehicle or with MN-exo, but not in brain slices from mice treated with NSC-exo. N = 6 mice/group (2 slices per mouse). *p < 0.05 two-tailed T-test. (PPTX 433 kb) [file 13024_2019_322_MOESM3_ESM.pptx]

## Slide 1
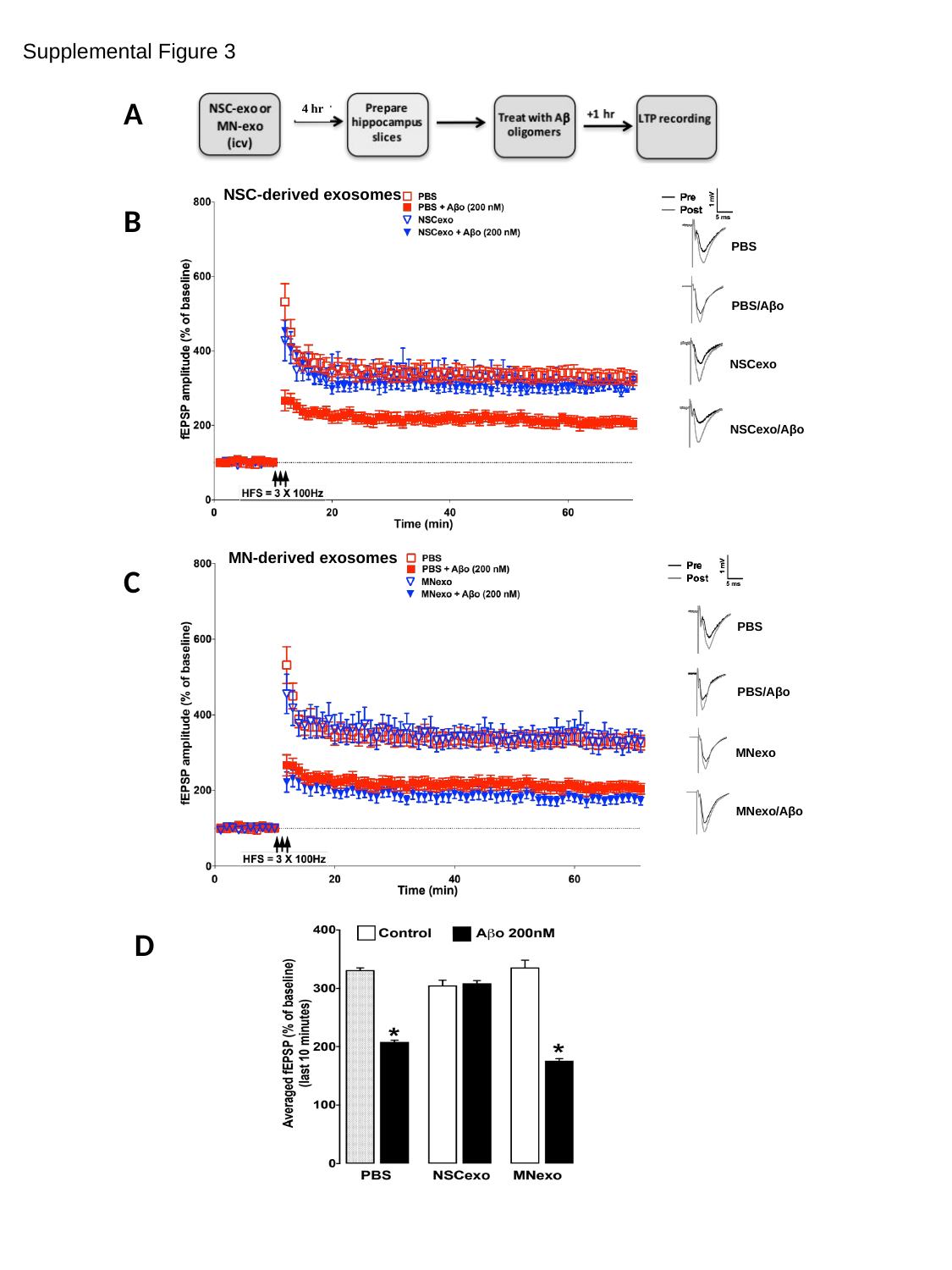

Supplemental Figure 3
A
4 hr
NSC-derived exosomes
PBS
PBS/Aβo
NSCexo
NSCexo/Aβo
B
MN-derived exosomes
PBS
PBS/Aβo
MNexo
MNexo/Aβo
C
D
