# Supplementary material for: Hippocampal stem cells promotes synaptic resistance to the dysfunctional impact of amyloid beta oligomers via secreted exosomes
Source: Mol Neurodegener. 2019 Jun 14;14:25. doi: 10.1186/s13024-019-0322-8 (PMC6570890; doi:10.1186/s13024-019-0322-8)
Supplement: Supplementary file 4 — Figure S4. Depletion of hippocampal neural stem cells following treatment of Nestin-δ-HSV-TK mice with valganciclovir. A) Construct scheme for Nestin-δ-HSV-TK transgenic mice. (B-G) Representative images of Nestin-δ-HSV-TK mice brain coronal sections showing the hippocampus dentate gyrus (B-E) and the subventricular zone (SVZ) of the lateral ventricle (F-G) stained with an antibody against green fluorescent protein (GFP, green) and neuronal nuclei (NeuN, red). GFP+ neural stem cells in the hippocampus dentate gyrus and SVZ are ablated after 4 weeks of Valganciclovir (VGCV) treatment (C, E, G) as compared to mice treated with vehicle (B, D, F). Calibration bar = 100 μm. (PPTX 1270 kb) [file 13024_2019_322_MOESM4_ESM.pptx]

## Slide 1
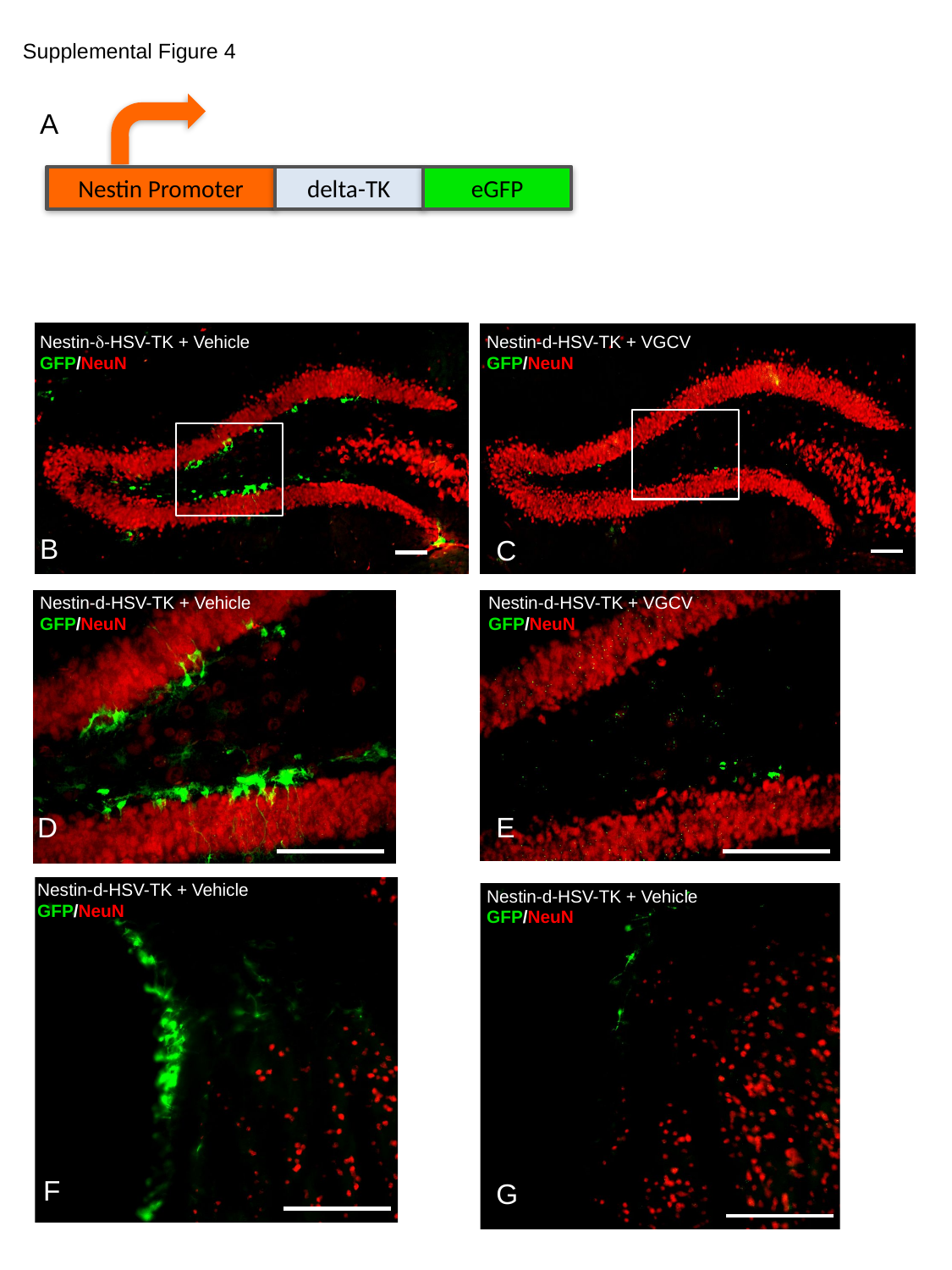

Supplemental Figure 4
A
Nestin Promoter
delta-TK
eGFP
Nestin-d-HSV-TK + Vehicle
GFP/NeuN
Nestin-d-HSV-TK + VGCV
GFP/NeuN
B
C
Nestin-d-HSV-TK + Vehicle
GFP/NeuN
Nestin-d-HSV-TK + VGCV
GFP/NeuN
SGL
D
E
Nestin-d-HSV-TK + Vehicle
GFP/NeuN
Nestin-d-HSV-TK + Vehicle
GFP/NeuN
F
G
