# Supplementary material for: Hippocampal stem cells promotes synaptic resistance to the dysfunctional impact of amyloid beta oligomers via secreted exosomes
Source: Mol Neurodegener. 2019 Jun 14;14:25. doi: 10.1186/s13024-019-0322-8 (PMC6570890; doi:10.1186/s13024-019-0322-8)
Supplement: Supplementary file 5 — Figure S5. Expression of NMDA and AMPA glutamate receptors in hippocampal synaptosomes. Total protein lysates of synaptosomes isolated from the hippocampus of mice injected ICV with PBS (vehicle), NSC-exo or MN-exo were analyzed by western blotting for the expression of total and phosphorylated glutamate AMPA (GluR1 and GluR2) and NMDA (NR1 and NR2) receptors (A). Band intensities were quantified using ImageJ software and normalized to β-actin. N = 3. *p < 0.05 Unpaired T-test. (PPTX 471 kb) [file 13024_2019_322_MOESM5_ESM.pptx]

## Slide 1
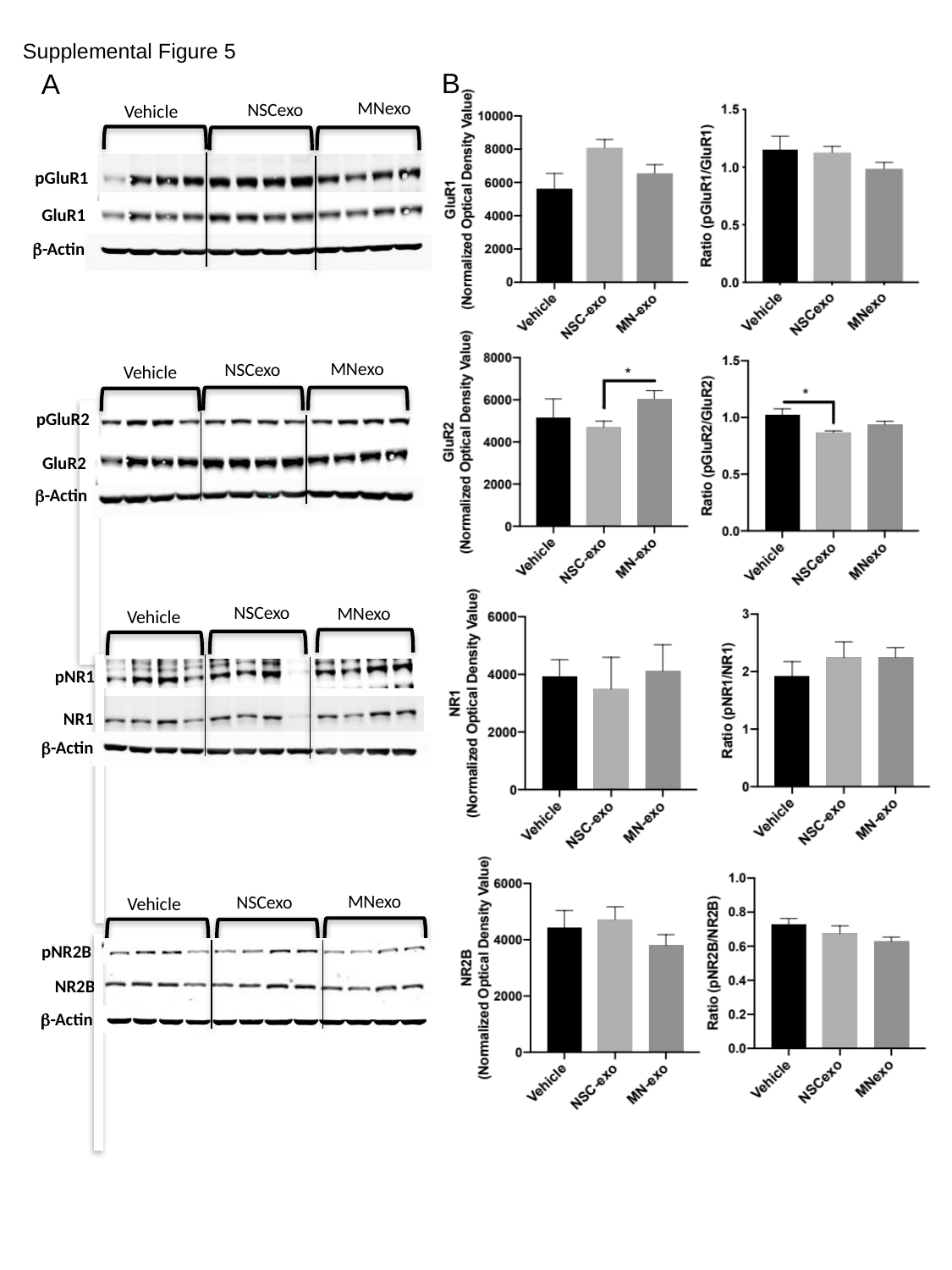

Supplemental Figure 5
B
A
MNexo
NSCexo
Vehicle
pGluR1
GluR1
b-Actin
MNexo
NSCexo
Vehicle
pGluR2
GluR2
b-Actin
NSCexo
MNexo
Vehicle
pNR1
NR1
b-Actin
MNexo
NSCexo
Vehicle
pNR2B
NR2B
b-Actin
