# Supplementary material for: Hippocampal stem cells promotes synaptic resistance to the dysfunctional impact of amyloid beta oligomers via secreted exosomes
Source: Mol Neurodegener. 2019 Jun 14;14:25. doi: 10.1186/s13024-019-0322-8 (PMC6570890; doi:10.1186/s13024-019-0322-8)
Supplement: Supplementary file 6 — Figure S6. Small RNA deep sequencing comparing RNA content in NSC-exo and MN-exo reveals that NSC-exo express a set of unique miRNAs involved in regulation of synaptic function and plasticity. A) Secreted exosomal miRNAs enriched in NSC-exo as compared to MN-exo. B) KEGG pathway analysis (P < 0.05) revealed potential target genes of these miRNAs enriched in pathways regulating synaptic function and plasticity. Each bar in blue indicates the number of miRNAs involved in the relevant pathway. The number of regulated genes involved in each pathway is indicated in parenthesis. Data is from 3 separate preparations from each cell type and 3 technical replicates. C) Mimics of miRNAs were injected ICV 24 h before sacrifice. The efficiency of the delivered mimics was confirmed by measuring levels of specific mRNAs regulated by the selected miRNAs, using RT-PCR. **P < 0.01; ***P < 0.001; ***P < 0.0001 vs. scrambled miRNA (T-test). N = 4 mice/group. (PPTX 230 kb) [file 13024_2019_322_MOESM6_ESM.pptx]

## Slide 1
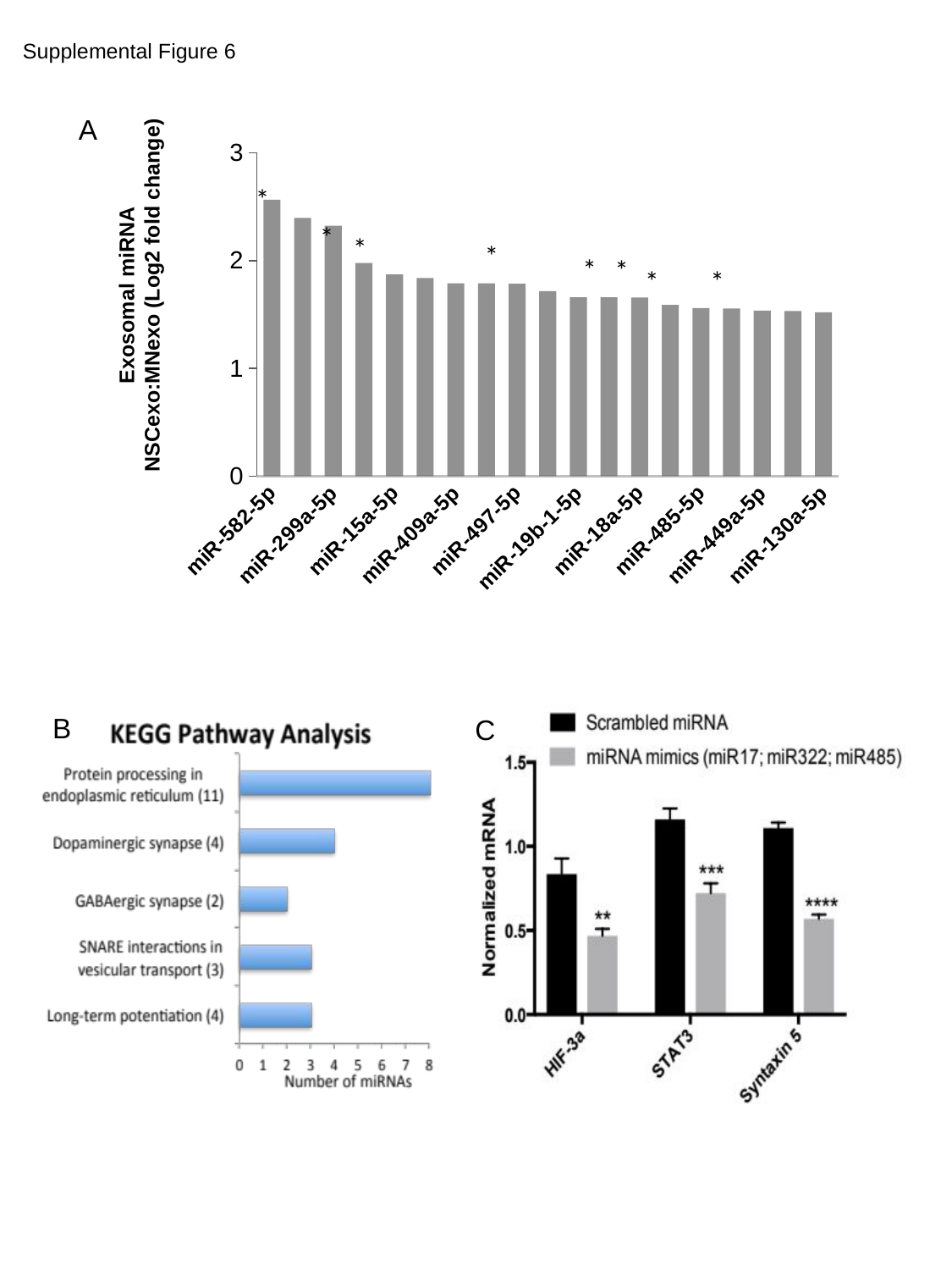

Supplemental Figure 6
A
### Chart
| Category | log2FoldChange |
|---|---|
| miR-582-5p | 2.564278929224177 |
| miR-322-5p | 2.394426622424858 |
| miR-299a-5p | 2.32215031861235 |
| miR-190a-5p | 1.97775739923732 |
| miR-15a-5p | 1.8708197100415 |
| miR-19a-5p | 1.83790395219846 |
| miR-409a-5p | 1.78940993978911 |
| miR-6215 | 1.78811527921846 |
| miR-497-5p | 1.78475333720541 |
| miR-329-5p | 1.71655359878742 |
| miR-19b-1-5p | 1.65997917596276 |
| miR-17-5p | 1.65926568954954 |
| miR-18a-5p | 1.65800131406011 |
| miR-15b-5p | 1.58903063011252 |
| miR-485-5p | 1.56 |
| miR-33-5p | 1.55461151790123 |
| miR-449a-5p | 1.5354167903247 |
| miR-449c-5p | 1.53153868036762 |
| miR-130a-5p | 1.51969221388391 |*
*
*
*
*
*
*
*
Exosomal miRNA
NSCexo:MNexo (Log2 fold change)
B
C
